# Establishment of a *Cuscuta campestris*‐mediated enrichment system for genomic and transcriptomic analyses of ‘*Candidatus* Liberibacter asiaticus’

**DOI:** 10.1111/1751-7915.13773

**Published:** 2021-03-03

**Authors:** Tao Li, Ling Zhang, Yunshuang Deng, Xiaoling Deng, Zheng Zheng

**Affiliations:** ^1^ Guangdong Province Key Laboratory of Microbial Signals and Disease Control South China Agricultural University Guangzhou, Guangdong 510642 China; ^2^ Citrus Huanglongbing Research Laboratory South China Agricultural University Guangzhou, Guangdong 510642 China

## Abstract

‘*Candidatus* Liberibacter asiaticus’ (CLas) is a phloem‐limited non‐culturable α‐proteobacterium associated with citrus Huanglongbing, a highly destructive disease threatening global citrus industry. Research on CLas is challenging due to the current inability to culture CLas *in vitro* and the low CLas titre in citrus plant. Here, we develop a CLas enrichment system using the holoparasitic dodder plant (*Cuscuta campestris*) as an amenable host to acquire and enrich CLas from CLas‐infected citrus shoots maintained hydroponically. Forty‐eight out of fifty‐five (87%) dodder plants successfully parasitized CLas‐infected citrus shoots with detectable CLas by PCR. Among 48 dodders cultures, 30 showed two‐ to 419‐fold CLas titre increase as compared to the corresponding citrus hosts. The CLas population rapidly increased and reached the highest level in dodder tendrils at 15 days after parasitizing citrus shoot. Genome sequencing and assembly derived from CLas‐enriched dodder DNA samples generated a higher resolution than those obtained for CLas from citrus hosts. No genomic variation was detected in CLas after transmission from citrus to dodder during short‐term parasitism. Dual RNA‐Seq experiments showed similar CLas gene expression profiles in dodder and citrus samples, yet dodder samples generated a higher resolution of CLas transcriptome data. The ability of dodder to support CLas multiplication to high levels, as well as its advantage in CLas genomic and transcriptomic analyses, make it an optimal model for further studies on CLas–host interaction.

## Graphical abstract

We develop a ‘*Candidatus* Liberibacter asiaticus’ (CLas) enrichment system using the holoparasitic dodder plant (*Cuscuta campestris*) as an amenable host to acquire and enrich CLas from CLas‐infected citrus shoots maintained hydroponically. The ability of dodder to support CLas multiplication to high levels, as well as its advantage in CLas genomic and transcriptomic analyses, make it an optimal model for further studies on CLas‐host interaction.

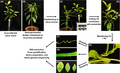

## Introduction

‘*Candidatus* Liberibacter asiaticus’ (CLas) is a fastidious α‐proteobacterium associated with citrus Huanglongbing (HLB, also known as yellow shoot disease), a destructive disease threatening citrus production worldwide (Jagoueix *et al*., 1994; Bové, 2006). Due to the current inability to culture *in vitro*, research on CLas has been mainly relied on analyses of CLas‐infected host materials. For instance, CLas‐infected citrus leaves midribs and their total DNA extracts were used for genome sequencing analysis of CLas (Garnier and Bové, 1983; Duan *et al*., 2009; Hartung *et al*., 2010; Zhang *et al*., 2011). Another challenge of CLas research is its uneven distribution and low titre in diseased citrus plants (Tatineni *et al*., 2008; Li *et al*., 2009). For these reasons, efforts have been made to obtain host sample with high CLas concentration by the assistance of others amenable plant hosts, such as periwinkle (*Catharanthus roseus*) (Zheng *et al*., 2014a).

Dodder (*Cuscuta* spp.) is a parasitic plant that can form the haustoria to uptake nutrients from the host plant through its haustoria (Kim and Westwood, 2015). The dodder plant was able to transmit the phloem‐limited fastidious microbes, e.g. virus, phytoplasma and bacteria, between source and receptor plants (Bennett, 1940; Kunkel, 1952; Ke *et al*., 1986; Tang and Fan, 1987; Zhou *et al*., 2007). Early studies had demonstrated that CLas can be transmitted by dodder from citrus to others citrus plants or new hosts, like periwinkle (*Catharanthus roseus*) and *Murraya paniculata* (Garnier and Bové, 1983; Ke *et al*., 1986; Tang and Fan, 1987; Zhou *et al*., 2007). In addition to be a microbial transmission tool, the dodder was found to support the multiplication of CLas to high levels (Hartung *et al*., 2010).

High‐throughput sequencing has now been commonly used for research on CLas, especially in genomic analyses (Duan *et al*., 2009; Lin *et al*., 2013; Katoh *et al*., 2014; Zheng *et al*., 2014a, 2014b, 2015, 2018; Wu *et al*., 2015a, 2015b; Kunta *et al*., 2017; Li *et al*., 2020). To obtain high quality CLas genome sequence, samples with high CLas titre are required. The insect vector (Asian citrus psyllid, *Diaphorina citri*) samples with high CLas titre were initially selected for CLas genome sequencing (Duan *et al*., 2009). However, it is not common to obtain the psyllid with high titre of CLas in the field (Ukuda‐Hosokawa *et al*., 2015), even under artificial feeding conditions in laboratory (Wu *et al*., 2018). Furthermore, high genome sequence variation was observed in CLas strains during psyllid transmission (Katoh *et al*., 2015). For plant hosts source, the low titre of CLas in samples affected the CLas genome sequencing. Therefore, a process of bacterial DNA enrichment was applied in CLas‐infected periwinkle and citrus plants samples to increase the ratio of CLas DNA to total DNA and further improve the CLas genome quality (Zheng *et al*., 2014a, 2014b, 2015, 2018). In addition, CLas could grow to high titre (Hartung *et al*., 2010) in dodder, but the use of dodder enrichment for CLas genomic research has not yet been explored.

Another interesting topic is the interaction between CLas and its host. This can be primarily achieved by profiling *in planta* genome‐wide transcriptome of CLas. However, few studies have been reported CLas transcriptome profiling by using RNA‐Seq, mainly due to low abundance of CLas RNA compared with host RNA. A recent study analysed in citrus and psyllids CLas expression profiles using RNA‐Seq, but the analysis lacked enough depth because CLas reads were minoritary (Zuñiga *et al.,* 2020). Therefore, to gain insight into CLas–host interaction during infection, higher resolution of *in planta* CLas transcripts is required.

Here, we developed a rapid CLas enrichment system using the dodder (*Cuscuta campestris*) to acquire and enrich CLas from infected citrus shoots. The efficiency of CLas enrichment by dodder was calculated based on CLas quantification. Genome sequencing of CLas strains from citrus shoots and the corresponding parasitized dodder samples were performed to evaluate whether CLas‐enriched dodder samples can be used as amenable host sources for CLas genomic study. We compared the genome quality and analysed the possible genomic variation of CLas strains in citrus shoots and the parasitized dodder. To investigate whether there were others microbe can also be transmitted simultaneously with CLas by dodder, the composition of microbial community in CLas‐infected citrus shoots and dodder was analysed by metagenomic analysis. In addition, the dual RNA‐Seq was generated to compare transcriptome profiling of CLas in citrus and the parasitized dodder. Our data showed that the CLas‐enriched dodder can be used as an optimal host for CLas genomic and transcriptomic analyses as compared to the citrus host.

## Result

### Appearance of dodder grown on hydroponic CLas‐infected citrus shoots

**Fig. 1:**
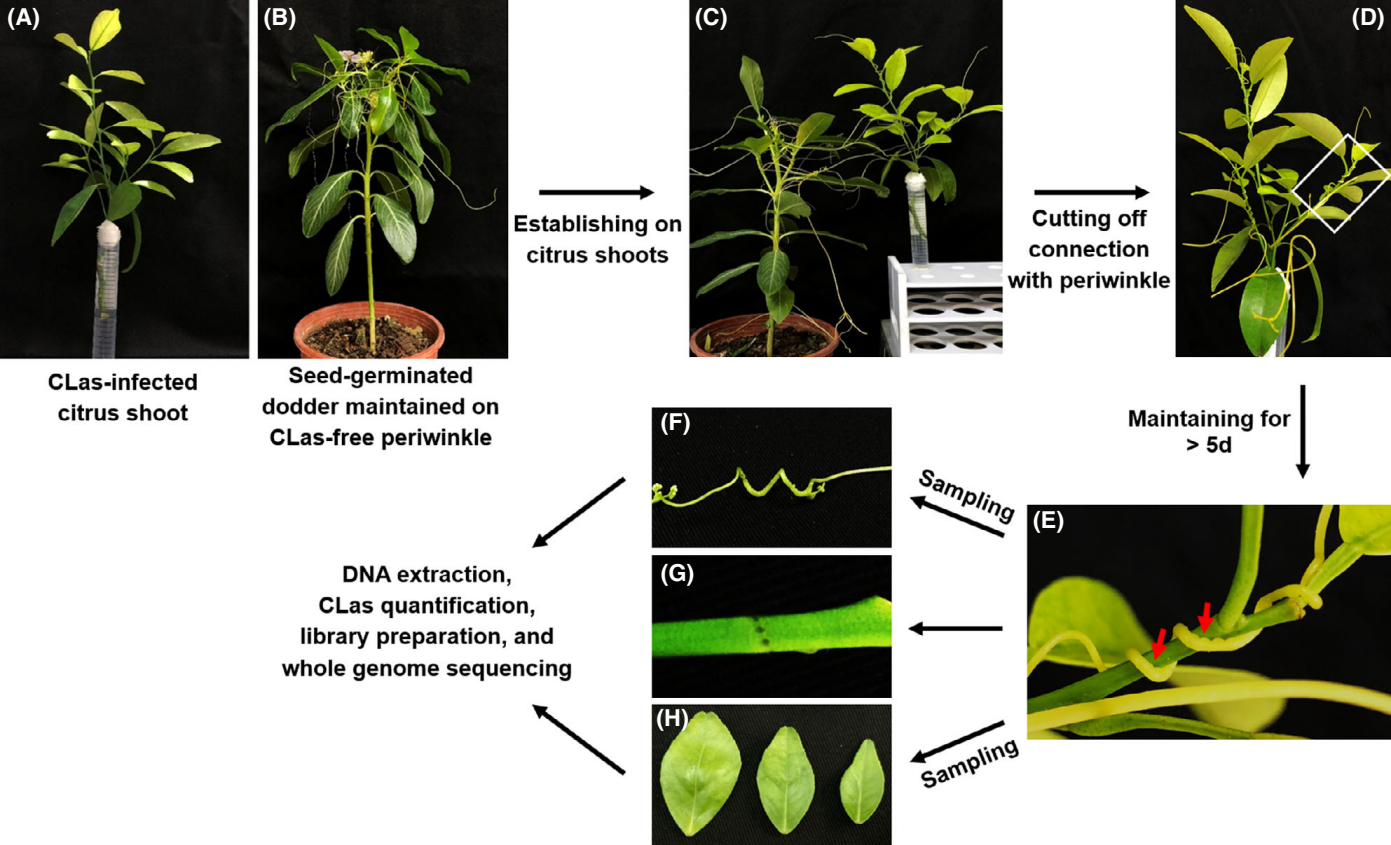
Procedure of dodder‐mediated enrichment of ‘*Candidatus* Liberibacter asiaticus’ (CLas) from excised CLas‐infected citrus shoot. A. CLas‐infected citrus shoot showing mottling and yellowing. B. the seed‐germinated dodder grown on CLas‐free periwinkle. C. the dodder tendrils started to parasitize in the CLas‐infected citrus shoot. D. the connection between dodder and periwinkle was immediately cut off after the dodder successfully formed the haustoria and parasitized in citrus shoot. E. the close‐up of white region in D. Note the dodder successfully formed the haustoria (red arrow) in citrus shoot. F. The dodder plant was sampled for DNA extraction; G, the wound in citrus shoot caused by dodder haustoria; H, three citrus leaves that closed to the parasitized site were sampled for DNA extraction.

**Fig. 2:**
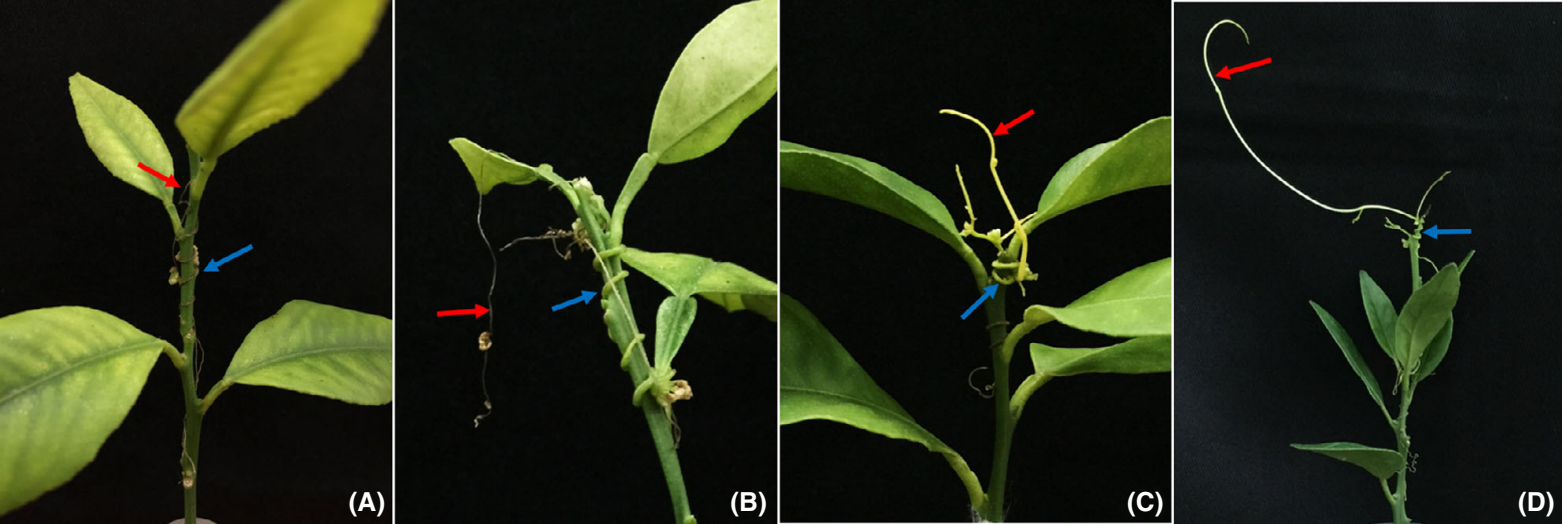
Four representative types of appearance of dodder grow on ‘*Candidatus* Liberibacter asiaticus’ (CLas)‐infected citrus shoots. The haustoria/coils of dodder was marked as blue arrow, and the tendrils (or growing point) were marked as red arrows. A, B, C and D represent Type I, Type II, Type III and Type IV appearance of dodder respectively. A. The dodders (including tendrils, coils and haustoria) rapidly declined within three days. B. the growing point or tendrils of dodder rapidly declined at early infesting stage, but the coils and haustoria were still robustly attached in citrus shoots. C. no decline of dodder was observed at early infesting stage, but dodder tendrils stopped elongating and the colour of dodder tendrils turned into dark yellow or orange after cutting off the connection with periwinkle. D. the dodder tendrils were able to keep elongating on the infected citrus shoot.

The CLas‐free dodder was initially maintained on healthy periwinkle and then used to infest hydroponic CLas‐infected citrus shoots (Fig. 1). Four representative types of dodder’s growth were observed (Fig. 2). In Type I, dodders tendrils, coils and haustoria rapidly declined in three days, these samples were discarded and CLas quantitation analysis was not performed (Fig. 2A). In Type II (Fig. 2B), dodder rapidly declined at early infesting stage, but the coils and haustoria remained alive for over seven days. Only the viable coils and haustoria of Type II dodder were collected for DNA extraction. In Type III (Fig. 2C), dodder continued to grow but the tendrils stopped elongating after cutting off the connection with periwinkle. The tendrils were maintained without decline between 8 and 24 days. In Type IV (Fig. 2D), dodder continued growing up to 24 days.

We observed that of the 55 dodder cultures, seven (13%) belonged to Type I, 21 (38%) were Type II, 17 (31%) were Type III and 10 (18%) were Type IV (Table 1). Except for Type I, the others 48 dodder plants (48/55, 87%) successfully formed the haustoria and parasitized CLas‐infected citrus shoots.

**Table 1:** Survival number and period of dodder plants grow on ‘*Candidatus* Liberibacter asiaticus’‐infected citrus shoots and quantitative comparison of ‘*Ca*. L. asiaticus’ population in citrus shoots and dodders.

Assay	Citrus varieties	No. of experiment set	Successfully parasitized	Survival period of dodder (days)^a^	Appearance of dodder^b^				Quantitative comparison of CLas (No. of experiment sets)	
					Type I	Type II	Type III	Type IV	Citrus > dodder	Citrus < dodder
I	*Citrus reticulata Blanco* cv. Gongkan	16	9 (56.3%)	11–14	7	7	1	1	6	3
II	*Citrus reticulata Blanco* c*v*. Shatangju	17	17 (100%)	5–24	0	6	7	4	6	11
III	*Citrus limon*	12	12 (100%)	17–20	0	6	5	1	4	8
IV	*Citrus reticulata Blanco* cv. Nianju	10	10 (100%)	16–18	0	2	4	4	2	8
	(Total)	55	48 (87.3%)	5–24	7	21	17	10	18	30

a Only the survival dodders were count for the survival period. A–B, where A and B represent the minimum and maximum survival time in this assay respectively.

b Four representative types of appearances of dodder grown on CLas‐infected citrus shoot in corresponding to A‐D in Fig. 1.

### PCR quantitation of CLas in citrus shoots and dodders

**Fig. 3:**
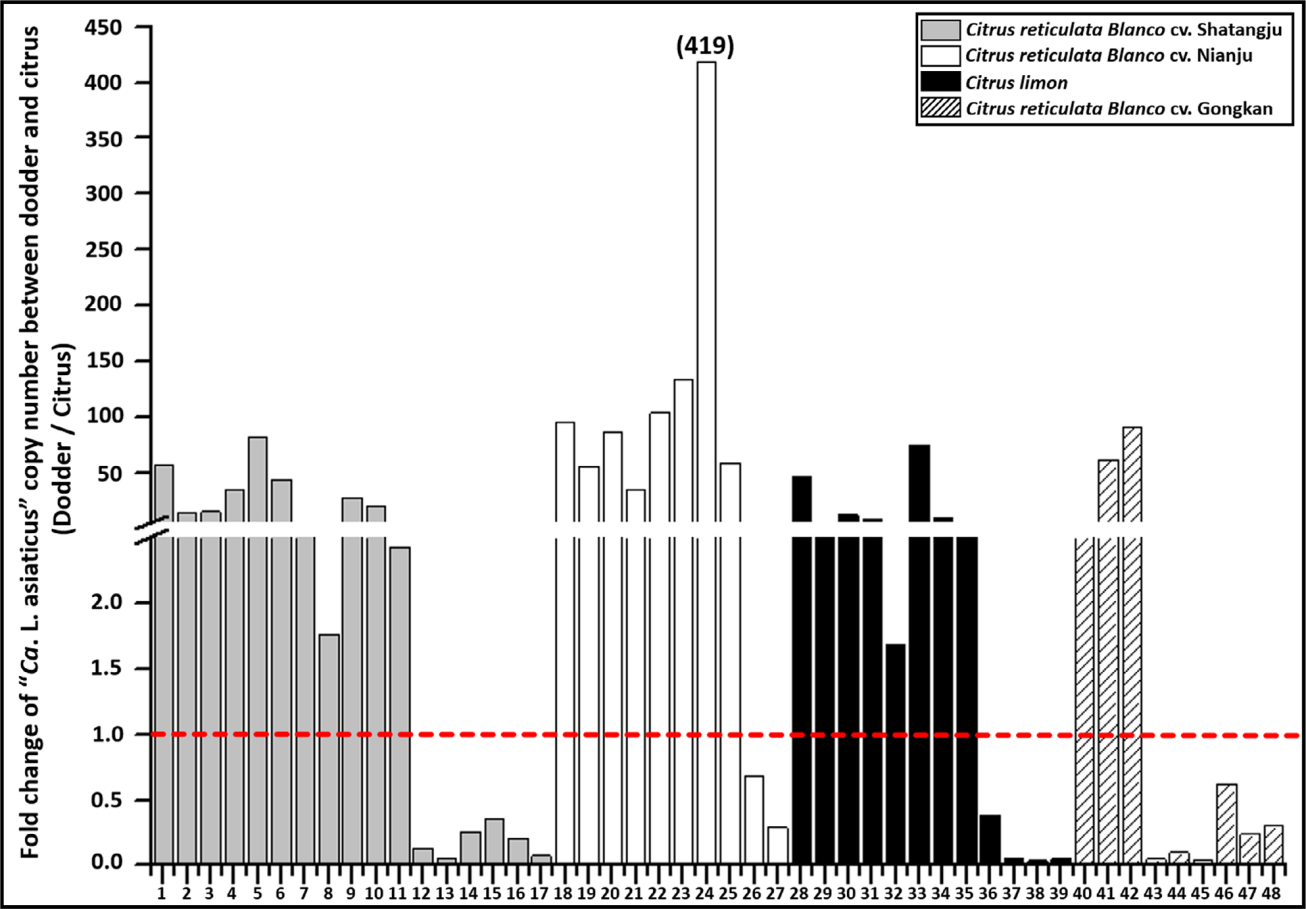
Enrichment fold of ‘*Candidatus* Liberibacter asiaticus’ (CLas) in citrus shoots and the parasitized dodder plant. The concentration of CLas was indicated as CLas cells/ng of total DNA. The number of citrus‐dodder sample sets was listed below the *x*‐axis. The enrichment fold = CLas concentration in dodder sample/CLas concentration in citrus sample.

In the hydroponic assays, we observed that all 48 successful parasitized dodder plants were positive for CLas, with concentration ranging from 42 to 834 072 cells per ng of total DNA. The higher concentration of CLas was observed in 30 dodder plants (62.5%) as compared to the corresponding citrus shoots. Of these 30 dodder plants, three dodders grew on *Citrus reticulata Blanco* cv. Tankan, 11 dodders grew on *Citrus reticulata Blanco* cv. Shatangju, eight dodders grew on *Citrus limon* and eight dodders grew on *Citrus reticulata Blanco* cv. Nianju (Table 1, Fig. 3). Compared to citrus shoots, the CLas concentration in the 30 dodders was enriched between two‐ and 419‐fold (Fig. 3).

**Fig. 4:**
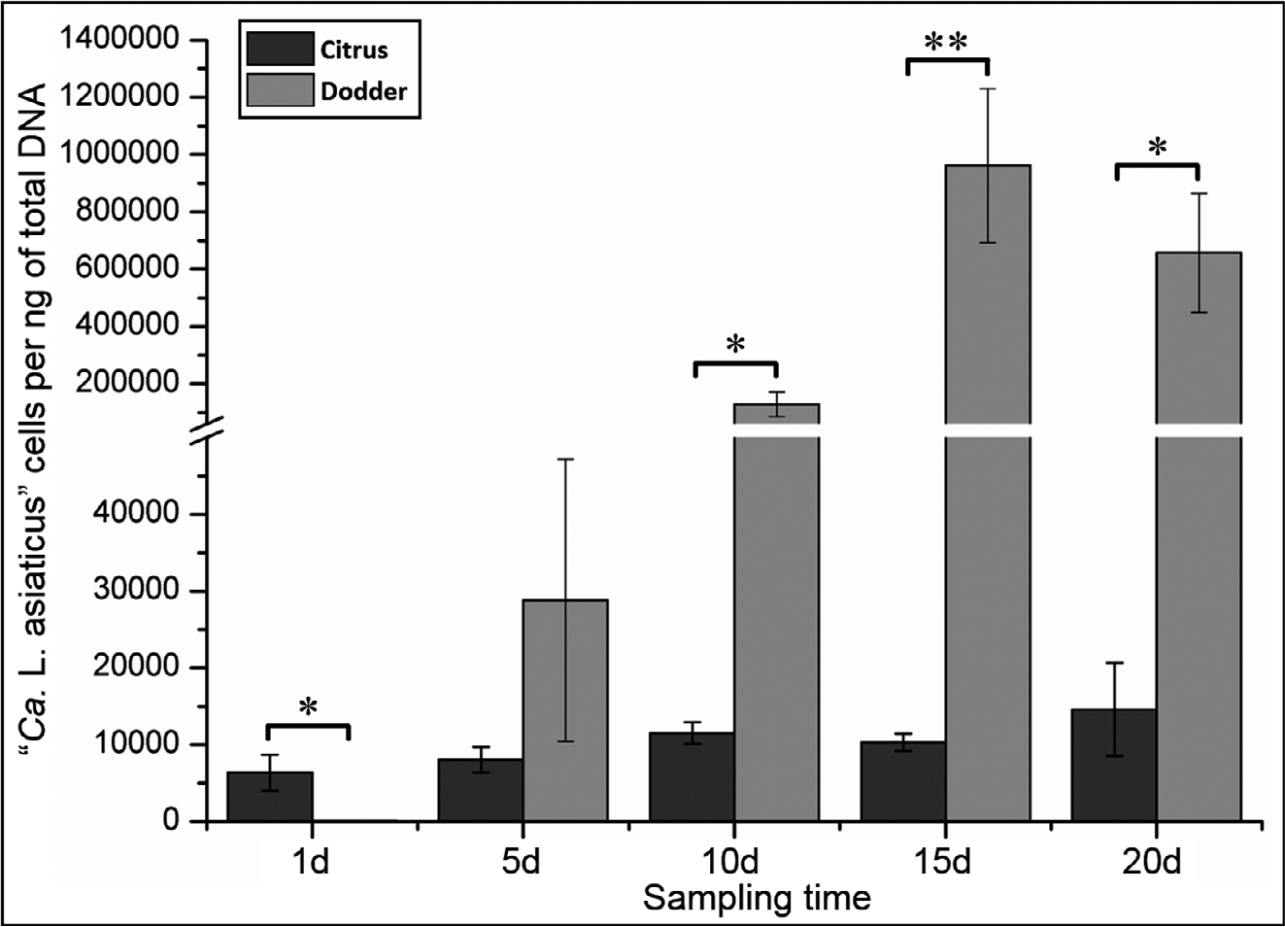
Quantification analysis of ‘*Candidatus* Liberibacter asiaticus’ (CLas) population in citrus shoots and the parasitized dodder plant during different parasitizing time. The concentration of CLas was indicated as CLas cells/ng of total DNA. ‘*’ = ‘*P* < 0.01’. ‘**’ = ‘*P* < 0.01’.

For the potted plant experiment, samples were monitored with time and it was observed that CLas population rapidly increased to a high level in the dodder after transmission from citrus plants (Fig. 4). The CLas concentration in dodder tendrils was low or even undetectable at day 1 (Fig. 4), then, increased rapidly and peaked at day 15 with an average of 9.6 × 10^5^ CLas cells per ng of total DNA (Fig. 4). In contrast, increase of CLas in citrus plants was significantly slower (Fig. 4). The CLas concentration in dodder tendrils was significantly higher than those in citrus shoots after 10 days (*P* < 0.05) (Fig. 4).

### Genome sequencing and comparison

Three sets of CLas‐infected citrus‐dodder samples (YNRL13, YNJS7 and GDHZ11 set) were selected for CLas genome sequencing (Table 2). Reads mapping to the chromosomal region of CLas strain A4 (CP010804.2) showed that the ratio of CLas reads/total reads in the dodder HiSeq data increased by 33.2‐fold, 5.5‐fold and 6.1‐fold with respect to those of citrus HiSeq data in YNRL13, YNJS7 and GDHZ11 set respectively (Table 2). Mapping to prophage sequences (SC1, SC2 and P‐JXGC‐3) showed consistent results between citrus and dodder samples (Table 3). The *de novo* assembly of dodder HiSeq data generated a higher quality CLas genome with fewer number of CLas contigs and longer N50 as compared to assembly result from citrus HiSeq data (Table 2). In addition, no genomic variation was found in CLas strains from citrus shoots and the parasitized dodder (Fig. S1).

**Table 2:** Summary of reference‐based assembly and *de novo* assembly of Hiseq data from ‘*Candidatus* Liberibacter asiaticus’‐infected citrus and dodder samples.

No.	Strain ID^a^	Host	Quantitative Real‐time PCR^b^		Referenced assembly^c^				De novo assembly		
			Copy number of CLas (cells/ng of total DNA)	Enrichment fold	Total reads	CLas reads	Percentage of CLas reads	Enrichment fold	No. of CLas contigs	N50 (bp)	Average length (bp)
1	YNRL13‐C	Lemon	11 218	74.5	87 833 502	76 791	0.09%	33.2	268	7059	4561
	YNRL13‐D	Dodder	834 072		66 985 762	2 005 475	2.99%		30	150 125	41 114
2	YNJS7‐C	Lemon	15 911	9.1	78 756 740	105 941	0.13%	5.5	131	17 065	9374
	YNJS7‐D	Dodder	144 690		71 334 076	513 845	0.72%		30	157 037	40 982
3	GDHZ11‐C	Shatangju	33 815	20.2	74 970 924	78 548	0.10%	6.1	197	9646	6126
	GDHZ11‐D	Dodder	683 739		85 282 104	521 362	0.61%		31	116 428	39 189

a N‐C: N was the name of sample. N‐C represented the CLas strain collected from citrus leave sample and N‐D represented the CLas strain collected from dodder sample.

b The quantitative Real‐time PCR analysis of CLas was determined with primer‐probe sets CLas‐4G/HLBp/HLBr (Bao *et al*., 2020). The concentration of CLas was considered as CLas cells/ng of total DNA. The enrichment fold = CLas concentration in dodder sample/CLas concentration in citrus sample.

c The chromosomal region of CLas strain A4 (CP010804.2, nucleotide position from 1 to 1 191 963) was used as reference. Enrichment fold = Percentage of CLas reads in dodder Hiseq data/percentage of CLas reads in citrus Hiseq data.

**Table 3:** Reads mapping result of citrus and dodder Hiseq data referenced to ‘*Candidatus* Liberibacter asiaticus’ chromosomal region and three prophages sequences.

No.	Strain ID^a^	Consensus size (bp, coverage X)^b^				Ratio (prophage/Chromosome)			Prophage type
		Chromosomal region	Type 1 prophage	Type 2 prophage	Type 3 prophage	Type 1 prophage/Chromosome	Type 2 prophage/Chromosome	Type 3 prophage/Chromosome	
1	YNRL13‐C	1 185 172 (9.7 X)	39 449 (10.4 X)	38 815 (9.7 X)	NF	1.08	1.00	NF	Type 1 + Type 2
	YNRL13‐D	1 187 573 (253.3 X)	39 997 (264.6 X)	38 844 (288.8 X)	NF	1.04	1.14	NF	Type 1 + Type 2
2	YNJS7‐C	1 185 755 (13.4 X)	38 165 (13.5 X)	NF	31 097 (12.0 X)	1.01	NF	0.9	Type 1 + Type 3
	YNJS7‐D	1 186 896 (64.9 X)	38 364 (70.1 X)	NF	31 188 (67.2 X)	1.08	NF	1.04	Type 1 + Type 3
3	GDHZ11‐C	1 186 170 (10.2 X)	NF	38 669 bp (10.1 X)	NF	NF	0.99	NA	Type 2
	GDHZ11‐D	1 187 392 (68.2 X)	NF	38 818 bp (68.8 X)	NF	NF	1.01	NA	Type 2

^a^ N‐C: N was the name of sample. N‐C represented the CLas strain collected from citrus leave sample and N‐D represented the CLas strain collected from dodder sample.

^b^ The chromosomal region of CLas strain A4 (CP010804.2, nucleotide position from 1 to 1 191 963) was used as reference. Type 1 prophage = SC1 (NC_019549.1), Type 2 = SC2 (NC_019550.1) and Type 3 = P‐JXGC‐3 (KY661963.1). The average coverage of prophage region was counted based on the prophage type‐specific region. The prophage type‐specific region was identified according to Zheng *et al*., 2018. NF represented the corresponding type of prophage had not been found, i.e. no unique read mapped to the specific region of the corresponding prophage.

### Metagenomic analysis of CLas‐infected citrus shoots and the parasitized dodders

In addition to CLas, the citrus chlorotic dwarf associated virus (CCDaV) and ‘*Candidatus* Portiera aleyrodidarum’ (CPa) were identified in both citrus shoot and dodder from YNRL13 set. Reads‐based abundance analysis showed only CLas was enriched in YNRL13 dodder sample (Table 4). For the others two sets (GDHZ11 and YNJS7), CLas was the most abundant (> 0.1% of total classified reads) microorganism found in citrus shoot and dodders. Analysis of taxa‐specific qPCR in others 27 citrus/dodder sets identified CPa in only six citrus shoots and CCDaV in ten citrus shoots and their parasitized dodders (Table 4, Table S1). However, among ten CCDaV‐positive citrus‐dodder sample sets, no enrichment of CCDaV was observed in the parasitized dodder (Table S1). For instance, CCDaV was identified in a high concentration in six citrus shoots (with *C*_t_ values ranged from 12 to 15) but in a lower concentration in their parasitized dodder tendrils (with *C*_t_ values ranged from 22 to 29) (Table S1).

**Table 4:** Metagenomic analysis of ‘*Candidatus* Liberibacter asiaticus’‐infected citrus shoots and the parasitic dodders.

HiSeq data based metagenomic analysis (ID‐host/reads)	‘*Candidatus* Liberibacter asiaticus’	Citrus chlorotic dwarf associated virus	*‘Candidatus* Portiera aleyrodidarum’
YNRL13‐Citrus	66 788	40 628	2158
YNRL13‐Dodder	1 761 766	15	1482
YNJS7‐Citrus	91 683	NF	NF
YNJS7‐Dodder	445 192	NF	NF
GDHZ11‐Citrus	66 937	NF	NF
GDHZ11‐Dodder	454 326	NF	NF
Taxa‐specific PCR result (No. of sets)			
Citrus+/Dodder+	27	10	0
Citrus+/Dodder‐	0	0	6
Citrus‐/Dodder‐	0	17	21
Citrus−/Dodder+	0	0	0
Total	27	27	27

NF, Not found.

### Efficiency evaluation for CLas gene expression profiling

**Fig. 5:**
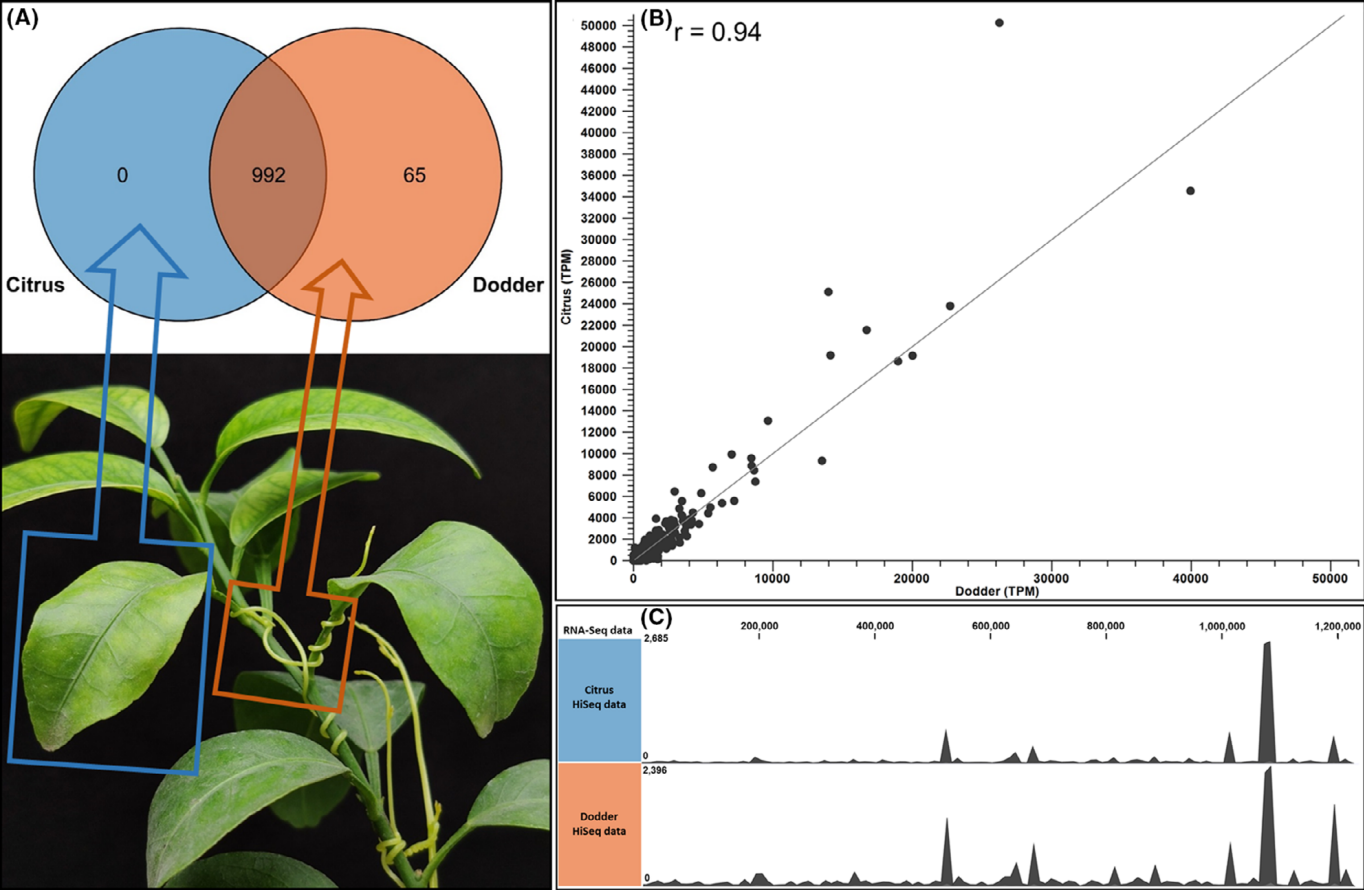
Genome‐wide gene expression profiling of ‘*Candidatus* Liberibacter asiaticus’ (CLas) in citrus shoot and its parasitized dodder. A. Venn diagram depicting the specifically and commonly expressed CLas genes in citrus shoot and its parasitized dodder plant. B. Scatter plot of CLas gene expression values (TPM, Transcripts Per Kilobase Million) derived from citrus and dodder RNA‐seq data. The figure is based on the Poisson distribution method. C. Whole genome‐wide reads mapping track of citrus and dodder RNA‐Seq in reference to CLas strain A4 (CP010804.2). Reads mapping was performed with CLC Genomic workbench v9.5 (length faction = 0.95; similarity fraction = 0.95). For each mapping result, the grey graph indicated reads coverage. The reads coverage was the average number of reads that align to a given position in the mapping. Note the similar reads coverage track (similar gene expression pattern) in citrus RNA‐Seq data and dodder RNA‐Seq data in reference to CLas strain A4 genome.

Illumina HiSeq generated a total of 1.58 × 10^8^ and 1.25 × 10^8^ reads for citrus and dodder RNA samples respectively. Mapping to CLas A4 genome (CP010804.2) and prophage sequences (SC1, SC2 and P‐JXGC‐3) identified a total of 55 688 CLas reads in citrus HiSeq data by generating a consensus of 1 021 604 bp with ~ 6.5× coverage, while a total of 137 795 CLas reads were identified in dodder HiSeq by generating a consensus of 1 208 490 bp with ~ 16.2 X coverage. A total of 992 CLas genes (88.2% of total genes) were expressed in both citrus shoot and dodder. Sixty‐five CLas genes (5.8% of total genes) were only expressed in dodder tendrils but at a low expression level (Fig. 5A, Table S3). It should be noted that a relative high correlation (*r* = 0.94) in expression levels of 992 CLas genes was observed between citrus and dodder RNA‐Seq data (evaluated by TPM values) (Fig. 5B). Additionally, genome coverage based on reads mapping also revealed similar genome‐wide gene expression pattern of CLas in citrus and dodder samples (Fig. 5C).

### Comparative transcriptome analyses of CLas in citrus and the parasitized dodders

Comparative transcriptome analyses identified 27 DEGs with fold change at a moderate level (Log2 fold change ranging from −2.15 to 1.55) (Table 5). Of the 27 DEGs, 26 genes were up‐regulated in citrus leaves and only one gene was up‐regulated in dodder tendrils (Table 5). Genes involved in lipid transport and metabolism were most abundant (four gene transcripts) among up‐regulated CLas genes in citrus compared to dodder (Table 5). Conversely, the 16S rRNA gene was the only identified DEG that was up‐regulated in dodder compared to citrus (Table 5).

**Table 5:** Differentially expressed genes of ‘*Candidatus* Liberibacter asiaticus’ between citrus HiSeq data and dodder HiSeq data.

No.	Gene Symbol^a^	Gene Name	Log2 fold change (dodder/citrus)^b^	Annotation	Function classification
1	CD16_RS01425	CD16_RS01425	−1.55	Accessory factor UbiK family protein	Not classified
2	CD16_RS02155	uppS	−1.44	di‐trans, poly‐cis‐decaprenylcistransferase	Lipid transport and metabolism
3	CD16_RS02880	CD16_RS02880	−1.44	Non‐canonical purine NTP pyrophosphatase	Nucleotide transport and metabolism
4	CD16_RS03415	flgK	−1.37	Flagellar hook‐associated protein FlgK	Cell motility
5	CD16_RS00625	rpmC	−1.32	50S ribosomal protein L29	Translation, ribosomal structure and biogenesis
6	CD16_RS02210	CD16_RS02210	−1.28	Hypothetical protein	Function unknown
7	CD16_RS04785	sufA	−1.22	Fe‐S cluster assembly scaffold SufA	Function unknown
8	CD16_RS01050	CD16_RS01050	−1.18	SDR family NAD(P)‐dependent oxidoreductase	Lipid transport and metabolism
9	CD16_RS01535	argF	−1.15	Ornithine carbamoyltransferase	Amino acid transport and metabolism
10	CD16_RS04585	CD16_RS04585	−1.15	Type 2 isopentenyl‐diphosphate Delta‐isomerase	Energy production and conversion
11	CD16_RS00685	rpsG	−1.13	30S ribosomal protein S7	Translation, ribosomal structure and biogenesis
12	CD16_RS02080	CD16_RS02080	−1.13	GtrA family protein	Function unknown
13	CD16_RS04575	CD16_RS04575	−1.13	GHMP kinase	Lipid transport and metabolism
14	CD16_RS04700	CD16_RS04700	−1.13	AprI/Inh family metalloprotease inhibitor	Cell wall/membrane/envelope biogenesis
15	CD16_RS05130	CD16_RS05130	−1.13	Hypothetical protein	Function unknown
16	CD16_RS03380	CD16_RS03380	−1.11	Flagellin	Cell motility
17	CD16_RS00165	CD16_RS00165	−1.10	Hypothetical protein	Not classified
18	CD16_RS02590	CD16_RS02590	−1.09	Hypothetical protein	Not classified
19	CD16_RS05085	CD16_RS05085	−1.06	Thiamine diphosphokinase	Nucleotide transport and metabolism
20	CD16_RS03045	pgsA	−1.06	CDP‐diacylglycerol‐‐glycerol‐3‐phosphate 3‐phosphatidyltransferase	Lipid transport and metabolism
21	CD16_RS05575	CD16_RS05575	−1.06	Hypothetical protein	Not classified
22	CD16_RS02160	CD16_RS02160	−1.03	CDP‐archaeol synthase	Function unknown
23	CD16_RS04260	CD16_RS04260	−1.03	Fihydrofolate reductase	Coenzyme transport and metabolism
24	CD16_RS04480	trmA	−1.03	tRNA (uridine(54)‐C5)‐methyltransferase TrmA	Translation, ribosomal structure and biogenesis
25	CD16_RS03940	CD16_RS03940	−1.02	Hypothetical protein	Function unknown
26	CD16_RS04420	CD16_RS04420	−1.02	Hypothetical protein	Not classified
27	CD16_RS01875	CD16_RS01875	2.16	16S ribosomal RNA	Function unknown

^a^ Gene symbol and gene name referenced to CLas A4 strain (CP010804.2).

^b^ Differentially expressed genes (DEGs) between citrus leaves HiSeq data and dodder HiSeq data were identified by GFOLD V1.1.4 with cut‐off values setting as Log2 Fold change≥│1│.

**Fig. 6:**
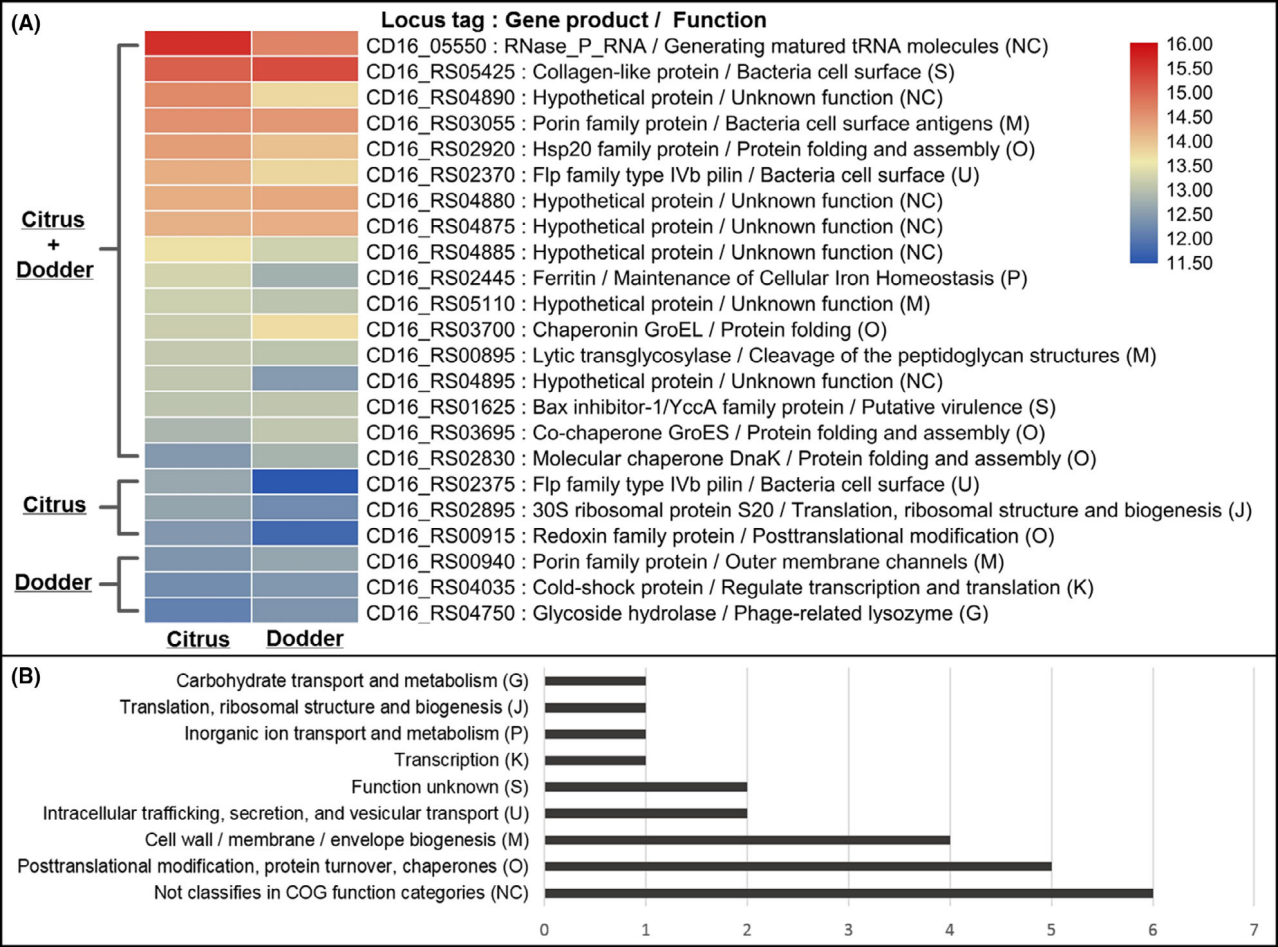
Functional classification of top 20 highly expressed genes of ‘*Candidatus* Liberibacter asiaticus’ (CLas) in citrus and the parasitized dodder. A. Heat map showed the log2 of normalized TPM (Transcripts Per Kilobase Million) values for top 20 most highly expressed CLas genes in citrus and the parasitized dodder. Genes belong to the list of top 20 most highly expressed genes in citrus or dodder were labelled in the left and bottom. The letter in the bracket was referencing to COG one letter code with detail descriptions showing in B. B. Summary of function classification for top 20 highly expressed CLas gene in citrus and dodder. The function classification of genes was performed with EggNOG v5.0 by using Clusters of Orthologous Groups (COG) database.

In addition, analysis of the top 20 most highly expressed genes in citrus shoot and dodders identified 17 CLas genes that encoded functions related to bacterial cell surface biogenesis and protein folding and assembly (Fig. 6). It was found that ribonuclease P (CD16_05550) and a collagen‐like protein (CD16_RS05425) were highly expressed CLas gene in citrus and dodder (Fig. 6). It was also found that a cluster of five genes (from CD16_RS04875 to CD16_RS04895) were highly expressed in both citrus and dodder, though the function of the corresponding products is at present unknown. Interestingly, two Flp family Type IVb pilin genes (CD16_RS02370 and CD16_RS02375), predicted to be involved in intracellular trafficking, secretion and vesicular transport, were also highly expressed in citrus shoot and dodder (Fig. 6).

## Discussion

Dodder has been used as a tool to transmit CLas from citrus to others citrus varieties or phylogenetically distant plants, such as periwinkle, or vice versa (Garnier and Bové, 1983; Ke *et al*., 1986; Hartung *et al*., 2010; Zhang *et al*., 2011; Zheng *et al*., 2014a). Hartung *et al*., (2010) carried out a detail study using dodder to study CLas, and they showed that CLas accumulated a high density in dodder tendrils. This was confirmed in our study as CLas density increased up to 419‐fold in dodder tendrils compared to the host citrus (Fig. 3). We have further taken advantage of the dodder enrichment capacity to obtain high density of CLas sample that facilitate genomic and transcriptomic analysis.

The use of hydroponic CLas‐infected citrus shoot as source for dodder parasitizing was simple and efficient (Fig. 1), although there were still some dodders that failed to enrich CLas from citrus shoot (Table 1). This was likely due to the failure of dodder to establish parasitism relationship with citrus shoots. CLas‐infection caused a severe phloem collapse and callose plugging, which block the phloem system and in turn inhibited the transport of photoassimilates (Kim *et al*., 2009; Etxeberria *et al*., 2009, 2012; Folimonova and Achor, 2010; Achor *et al*., 2010; Koh *et al*., 2012; Brodersen *et al*., 2014). Thus, for some citrus shoots whose phloem system was already under the destructive effect by CLas, the nutrition supplies did not satisfy the dodder nutrient requirements. Preliminary observations suggested that the use of young/viable citrus shoots could increase dodder survival rate.

Early studies used dodder as host source for CLas genome sequencing for reason that it contained less contaminating host chloroplast and mitochondrial DNA (Zhang *et al*., 2011). Our data, in addition, support another advantage of dodder in CLas genomic studies that is the improvement of CLas genomic data quality (Table 2, Table 3 and Fig. S1). However, we noted that there were still a very high ratio of host reads in dodder HiSeq data (Table 1), indicating high ratio of host DNA remained in the dodder total DNA. In a previous study (Zheng *et al*., 2014a), bacterial DNA in periwinkle host was enriched by removing the methylated host DNA using a microbial DNA enrichment kit. It will be of high interest to apply the same technique for further reduction of dodder DNA, i.e. to further enrich CLas in system reported here.

No genomic variation was found in CLas strains after transmission from citrus to dodder with short‐term parasiting. In previous studies, it was proposed that the variation of intracellular bacteria was mostly induced by host‐driven selective forces during the host–bacteria interaction (Toft and Andersson, 2010). A previous study also reported the changes in the variable number of tandem repeats in CLas genome via psyllid transmission (Katoh *et al*., 2015). In this study, CLas was transmitted from citrus to dodder and only maintained for a short period (≤ 24 days), which seems to be not long enough to induce variations. Nutrients for growth of dodder, as well as the growth of CLas, were all from the citrus host. Then, it is likely that the similar nutrient composition in dodder tendrils and the phloem of citrus shoot could also reduce the host‐driven selective forces for CLas and make the genetic variation much slower. In addition, the similar CLas gene expression patterns in citrus shoot and its parasitized dodder also suggested that similar CLas–host interaction in citrus and dodder took place (Fig. 6).

Few studies have reported the *in planta* genome‐wide transcriptome profiling of CLas, until a recent study that employed RNA sequencing for analyses of CLas expression profile by using phloem‐enriched samples from CLas‐infected citrus trees (Zuñiga *et al*., 2020). However, less than 0.53% of total reads were identified as CLas reads in RNA‐Seq data derived from CLas‐infected citrus phloem samples (Zuñiga *et al*., 2020). The use of CLas‐enriched dodder RNA sample in this study provided a higher resolution of CLas transcripts, and we observed similar gene expression patterns of CLas both in citrus host and dodders. Dodder was known to facilitate bidirectional movement of mRNAs between host and parasite (Kim *et al*., 2014). The CLas mRNA in dodder plant could be transferred from citrus host via the haustoria’s connection or transcribed by CLas cells in dodder tendrils after transmission from citrus shoot. A previous study had demonstrated that the dodder microRNA (miRNAs) can target the host mRNA and regulate the host gene expression during parasitism (Shahid *et al*., 2018). However, the similar CLas transcriptome profiling in citrus and dodder indicated that the CLas gene expression may have not been affected by the dodder plants. This is consonance with dodder obtaining not only nutrients but also metabolites and proteins from host plants (Kim and Westwood, 2015). Therefore, it is not surprising the CLas transcriptome profiles in dodder and citrus are similar because they share a similar micro‐environment that contained similar nutrients or even antimicrobial substance.

It should be noted that two Flp family Type IVb pilin genes (CD16_RS02370 and CD16_RS02375) were highly expressed in both citrus and dodder tendrils (Fig. 6). The Type IV pilis are dynamic adhesive filaments in the surface of bacteria involved in host cells adherence, DNA uptake, twitching motility and bacterial interactions (Maier and Wong, 2015; Craig *et al*., 2019). An early study demonstrated that a CLas Type IV pilin gene *flp3* was highly expressed in psyllid compared to in citrus plant and could be involved in attachment to the psyllid midgut (Andrade and Wang, 2019). In CLas‐infected citrus, a filamentous‐like material was observed in the surface of CLas cells and connected to the plasma membrane at sieve plate pore (Achor *et al*., 2020). Although it is still unclear whether these filaments are involved in CLas adherences to the plasma membrane and if they are Type IV pili.

In conclusion, we developed a rapid enrichment procedure by using dodder as an amenable host to capture and enrich CLas from the hydroponic excised CLas‐infected citrus. The use of CLas‐enriched dodder DNA samples generated higher quality CLas genome sequence data than those from citrus host. No genomic variation was observed in CLas strains after transmission from citrus shoots to dodder during short‐term parasitizing. Dual RNA‐Seq analyses of CLas‐enriched dodder samples generated a higher resolution CLas transcriptome data than those obtained in citrus host, and a similar CLas gene expression pattern was found in dodder and citrus. The dodder‐mediated CLas enrichment system can benefit both CLas genomic and transcriptomic research.

## Experimental procedures

### Plant material and water‐culture maintaining of citrus shoot

HLB‐affected citrus shoots that used for dodder’s parasitizing were collected from two citrus growing provinces in China, i.e. Guangdong and Yunnan provinces, between March 2018 and December 2018. A total of four citrus cultivars were collected, including Shatangju (*Citrus reticulata Blanco* cv. Shatangju), Lemon (*Citrus limon*), Gongkan (*Citrus reticulata Blanco* cv. Gongkan) and Nianju (*Citrus reticulata Blanco* cv. Nianju) (Table 1, Table S1). The symptomatic citrus shoots were kept moisturized before bring back to the laboratory from citrus orchards. The bottom of citrus shoot, about 1 cm in length, was further cut out. The remaining stem of young shoot was maintained with the distilled water in a 15 ml tube immediately and fixed with cotton to keep the leaves in the air (Fig. 1A). Three citrus leaves from each citrus shoot were sampled for DNA extraction and used for PCR detection of CLas. Citrus shoots, which were detected as CLas‐positive and can be maintained in distilled water over three days with most leaves still attached, were further used for the CLas enrichment experiment.

### Cultivation of dodder and CLas enrichment procedure

Dodder (*Cuscuta campestris*) was germinated from the seed and placed on CLas‐free periwinkle plant (*Catharanthus roseus*) (Fig. 1B). Tendrils from vigorously growing dodder were used to infest another CLas‐free periwinkle plant to get more dodder tendrils. Approximately a week after initial attachment, new tendrils emerged and elongated until to have 3–5 tendrils for each dodder. Thereafter, they can be used to parasitize the CLas‐infected citrus shoots. CLas enrichment was performed on both hydroponic and potted plant experiments.

For the hydroponic assay, each CLas‐infected citrus shoot was maintained hydroponically and parasitized by the tendrils from the individual dodder plant (Fig. 1C). After dodder successfully formed the haustoria and parasitized in citrus shoot, the connection between dodder tendrils and periwinkle was immediately cut off (Fig. 1D). The dodder tendrils and three citrus leaves (closed to the parasitizing site) were sampled simultaneously when the dodder tendrils/coils started to decline (e.g. the tendril started to wilt) (Fig. 1F, H). The survival period of dodder on citrus shoots was counted as the time starting at day that the connection between dodder and periwinkle was cut and ending at the day that dodder tendrils/coils began to decline.

For the potted plant experiment, six potted CLas‐infected two‐year citrus seedlings, which contained at least four symptomatic shoots, were used as receptor plants for dodder’s parasitizing. The CLas‐free dodder tendrils (grew on CLas‐free periwinkle) were used to infest the CLas‐infected citrus seedlings. The connection between dodder and periwinkle was cut off after the dodder successfully formatted the stable haustoria in citrus shoots/branches. As a control, the dodder tendrils and three citrus leaves near the parasitized site were collected at first day when cut off the connection with periwinkle. Therefore, both citrus leaves and dodder tendrils from same branch were sampled every five days until 20 days. Dodder was removed from citrus plants as needed if the dodder grew excessively and began to damage the host. All dodder and citrus leaves samples were immediately put into the liquid nitrogen when sampled and brought back to the laboratory for both DNA and RNA extraction.

### DNA extraction and CLas quantitation

For citrus leaves samples, total DNA was extracted from the leave midribs. For dodder samples, only fresh tissue (tendrils, coils or haustoria), which still showed green or light orange, were collected when it started to decline and used for DNA extraction. 100 mg of fresh tissue from citrus midribs or dodder was extracted by E. Z. N. A. HP Plant DNA Kit (OMEGA Bio‐Tek Co., Guangdong, China).

Quantitation of CLas was performed by TaqMan^®^ Real‐time PCR developed by Bao *et al*. (2020) with primer‐probe set, CLas4G/HLBp/HLBr. The plasmid PCLas4G, which harboured the CLas4G‐HLBr specific region, was constructed by cloning a 78‐bp fragment that was amplified using CLas4G/HLBr (Table S2) into *pEASY*‐T1 vector (TransGen Biotech Co., Beijing, China). A standard equation, *y* = −3.201*x* + 42.7 (*R* ^2^ = 0.999), was developed to quantify CLas copy number in each DNA sample. Briefly, the quantification of bacterial populations was considered as cells per nanograms of total DNA. The concentration of plasmid PCLas4G and each DNA sample was determined using Qubit (Thermo Fisher Scientific Inc., Waltham, MA, USA). The copy number of plasmid PCLas4G was calculated using the formula: number of copies = (amount in nanograms × Avogadro’s number)/(length in base pairs × 1 × 10^9^ × 650). The Avogadro’s number is 6.022 × 10^23^, and the average weight of a base pair is assumed to be 650 daltons. The quantitative TaqMan^®^ Real‐time PCR was performed in CFX Connect Real‐Time System (Bio‐Rad, Hercules, CA, USA). The TaqMan^®^ PCR reaction mixture contained 10 μl of Bestar^®^ qPCR Master Mix (DBI^®^ Bioscience, Shanghai, China), 1 μl of DNA template (~25 ng), 0.2 μl of PCR probe (10 μM), 0.4 μL of each forward and reverse primer (10 μM) in a final volume of 20 μl under the following procedure: 95°C for 2 min, followed by 40 cycles at 95°C for 10 s and 60°C for 30 s, with fluorescence signal capture at the end of each 60 °C step. A standard equation, *y* = −3.201*x* + 42.7 (*R* ^2^ = 0.999), was developed based on a previous study by Bao *et al*. (2020). All data were analysed using Bio‐Rad CFX Manager 2.1 software with automated baseline settings and threshold.

The enrichment fold of CLas concentration by dodder was calculated using the formula, enrichment fold = the copy number of CLas (cells/ng of total DNA) in dodder sample/the copy number of CLas (cells/ng of DNA) in the corresponding citrus samples. The copy number of CLas between citrus samples and the parasitized dodder tendrils was analysed by independent‐sample t‐test under the SPSS Statistic package (v19.0; IBM, Armonk, New York, NY, USA).

### Genome sequencing and assembly

Three sets of total DNA samples extracted from CLas‐infected citrus shoots and the parasitized dodders were selected for whole genome sequencing with Illumina HiSeq platform (Illumina, Inc., San Diego, CA, USA). Illumina sequencing was carried out by a commercial source. The citrus HiSeq data were initially mapped to the *Citrus sinensis* genome (AJPS00000000.1), *Citrus clementina* genome (AMZM00000000.1), *Citrus sinensis* mitochondrion genome (NC_037463.1) and *Citrus sinensis* chloroplast genome (DQ864733.1) by using Bowtie2 software (Langmead and Salzberg, 2012). For dodder HiSeq data, the *Cuscuta australis* genome (NQVE00000000.1) and *Cuscuta campestris* (OOIL00000000.1) were used as reference for reads mapping with Bowtie2 software (Langmead and Salzberg, 2012). All mapped reads were removed, and only the unmapped reads were retained for CLas assembly. A previously developed procedure (Zheng *et al*. 2016) was followed to perform *de novo* assembly and referenced‐based assembly. Briefly, the *de novo* assembly of CLas genome was conducted with Velvet 1.2.10 (kmer = 75, min_contig_lgth = 1000) (Zerbino and Birney, 2008). Contigs from *de novo* assembly were blast against with CLas strain A4 genome (CP010804.2) and three prophage sequences (SC1, NC_019549.1; SC2, NC_019550.1 and P‐JXGC‐3, KY661963.1) by using Standalone BLASTn software (word_size = 28, *e*‐value = 1e‐5) (Camacho *et al*. 2009). The hit contigs from blast result were identified as the candidate CLas contigs and retrieved. All candidate CLas contigs from *de novo* assembly were further used to blast against NCBI nucleotide database by web BLAST with the default setting. The chimeric contigs were removed based on the web BLAST result, and the rest contigs composed the draft genome sequence of CLas. The draft CLas genome was further used as reference to re‐mapped with HiSeq reads by using Bowtie2 software (Langmead and Salzberg 2012). All mapped reads were collected and designed as CLas reads.

For reference‐based assembly, the published CLas genomes (strain A4: CP010804.2, strain Psy62: CP001677.5 or strain JXGC: CP019958.1) and three prophage sequences (SC1: NC_019549.1, SC2: NC_019550.1 and P‐JXGC‐3: KY661963.1) were used as references to guide the assembly by CLC Genomic workbench 9.5 (length fraction = 0.95, similarity fraction = 0.95). The detail mapping report of each sequencing data was created, and the mapping consensus was extracted by CLC Genomic workbench 9.5. The total number of mapped reads of each HiSeq data was directly obtained from the mapping report. The average coverage of prophage region was counted based on mapping result with prophage type‐specific region, which was identified according to Zheng *et al*. (2018).

The initial version of CLas genome was obtained by combining with the assembly contigs from both *de novo* assembly and referenced‐based assembly based on sequence overlap using Standalone BLASTn software (word_size = 28, *e*‐value = 1e‐5). The optimized version of CLas genome was optimized by gap‐closure PCR and Sanger sequencing. Genome annotation was performed on the RAST server (http://rast.nmpdr.org/) (Aziz *et al*. 2008).

### Genome comparison of CLas from citrus and the parasitized dodder

Compared with the dodder‐origin CLas genome (named CLas‐D), the average coverage of CLas genome sequenced from citrus (named CLas‐C) was lower (< 15x). To evaluate the possible genomic variation of CLas strains after transmission from citrus to dodder, the high quality CLas‐D genome was used as reference for mapping with citrus HiSeq data by CLC Genomic Workbench v9.5 (length fraction = 0.95, similarity fraction = 0.95). The mapping result was further used for quality‐based variation detection by CLC Genomic workbench v9.5 (the required variant reads count was set as the average coverage of whole genome sequence) to identify the possible genomic variation between CLas‐D and CLas‐C genome. All possible variations (single nucleotide polymorphisms, SNPs or Insertion/Deletion, In/Del) were further confirmed by using the variant‐supported reads to blast against with both CLas‐C and CLas‐D genome through a web BLASTn‐based method. Briefly, when the variation‐contained reads can match the multiple copies loci in CLas chromosomal region or different types of prophage sequence with > 95% coverage and > 95% identity, the reads‐covered variation was identified as the false‐variation due to the high similar region between repeat loci in the CLas genome or among different types of prophages. The web BLASTn was performed with the default setting (expect threshold = 0.05, word size = 28).

### Metagenomic analysis

HiSeq data of three sets of CLas‐infected citrus shoots and their parasitized dodders were initially used for metagenomic analysis. All unmapped reads from dodder and citrus HiSeq data that retained after filtering with host genome (citrus or dodder) were used for metagenomic analysis with Kaiju software (Menzel *et al*., 2016). The NCBI non‐redundant protein database was used for taxonomic classification by Kaiju software. Only taxa that comprised at least 0.1% of total classified reads were considered for further analyses. Each unique taxa in the taxa list of each sample was filtered by removing the microbe which was known as pathogen of human/animal or the common contaminating microbe in DNA extraction and other laboratory regents (Salter *et al*., 2014). An *in silico* evaluation was performed to initially confirm the existence of each microbe in the taxa list. Briefly, the representative genome sequence for each filtered taxa was downloaded from NCBI Nucleotide database and used as reference for reads mapping with filter reads. The covered of each reference genome from mapping result was calculated. The taxa were regarded as the false‐taxa and removed from the taxa list if the covered of the corresponding reference genome and the consensus coverage were significantly low (less than 5% covered and 5× depth). In addition to *in silico* evaluation for each candidate taxa, the taxa‐specific Real‐time PCR primer set (Table S2) was designed based on consensus sequence (from mapping result) of each microbe by using Primer 3 software (Untergasser *et al*., 2012) and further used to test in 27 citrus‐dodder sample sets (including CLas‐enriched and non‐enriched sets). The possible transmission of each taxa between citrus and dodder was evaluated based on the PCR result.

### In planta genome‐wide gene expression of CLas in citrus and the parasitized dodder

To ensure the sufficient sequencing data for gene expression analysis of pathogen, the dual RNA‐Seq typically required a relatively large amount of host cells be infected by pathogen. Therefore, citrus leaves samples with a high CLas titre were selected for RNA extraction. The dodders tendrils with highest CLas concentration collected after 15 days’ parasitizing (Fig. 4), as well as three leaves from the host citrus shoot, were used for RNA extraction and dual RNA‐Seq analyses. Three biological replicates of citrus leaves and dodder tendrils samples were collected from different citrus/dodder experiment sets. The three replicates were further mixed as one sample for RNA extraction. RNA was extracted by using Plant RNA Kit (OMEGA Bio‐Tek Co.). The quality of total RNA sample was evaluated by Qubit 2.0 (Thermo Fisher Scientific Inc.) and Agilent 2100 (Agilent Technologies Inc., Santa Clara, CA, USA). The qualified RNA samples were further used for library preparation with a TruSeq RNA library Prep Kit (Illumina, San Diego, CA, USA) by removing rRNA from total RNA. Sequencing was carried out on an Illumina HiSeq 3000 system with 150‐bp paired‐end reads by a commercial source. All HiSeq reads from citrus leaves and dodder tendrils samples were mapped to CLas strain A4 genome (CP010804.2) by CLC Genomic workbench v9.5 (QIAGEN Bioinformatics, Aarhus, Denmark) (length faction = 0.95; similarity fraction = 0.95). Reads mapped to each CLas gene were then summarized into count tables of ‘Total Gene Reads’. The Transcripts Per Kilobase Million (TPM) method was used for normalization of RNA‐seq, i.e. TPM = A×10^6^ × 1/∑(A), where A = total reads mapped to gene × 10^3^/gene length in bp. Differentially expressed genes (DEGs) between citrus leaves HiSeq data and dodder HiSeq data were identified by GFOLD V1.1.4 with cut‐off values setting as Log_2_ Fold change ≥ │1│(Feng *et al*., 2012). Functional annotation and orthologs assignment of identified DEGs were performed by eggNOG‐mapper (Huerta‐Cepas *et al*., 2017). The top 20 most highly expressed CLas genes that evaluated by TPM value from citrus leaves data and dodder data were manually retrieved and compared. Heat map for comparison of CLas gene expression (by TPM value) between citrus leaves and dodder was generated in TBtools software (Chen *et al*., 2020). The gene‐specific Real‐time PCR for 20 selected DEGs was used to verify the gene expression result based on RNA‐Seq. All primers were listed in Table S2. RNA‐Seq data used in this study had been deposited in the NCBI Sequence Read Archive (SRA) database (BioProject: PRJNA675299 and Submission ID: SUB8484188).

## Funding Information

This work was supported by Special fund for the National Key Research and Development Program of China (2018YFD0201500), National Natural Science Foundation of China (31901844), the cultivating key project of international cooperation in science and technology (2019SCAUGH04) and Chinese Modern Agricultural Technology Systems (CARS‐26).

## Conflict of Interest

The authors declare that they have no conflict of interest.

## Author contributions

T. L., Z. Z. and X. D. conceived and designed the experiments. T. L., L. Z., Y. D. and Z. Z. performed the experiments. T. L. and Z. Z. contributed to bioinformatics and statistical analysis, prepared figures/table and wrote the draft manuscript. Z. Z. and X. D. reviewed and revised the manuscript.

## Supporting information


**Fig. S1**. Evaluation of the genomic variation of ‘*Candidatus* Liberibacter asiaticus’ by the quality‐based variation detection based on reads mapping from citrus samples and its parasitized dodder samples. Reads mapping was generated by using dodder‐origin CLas genome (including the chromosomal region, strain‐C and prophage region, P‐strain‐1, 2, or 3) as reference for mapping with citrus HiSeq data. The quality‐based variation detection of each read mapping was performed with CLC Genomic workbench v9.5.
**Table S1**. Quantification of ‘*Candidatus* Liberibacter asiaticus’ in citrus and the parasitized dodder and taxa‐specific PCR result.
**Table S2**. General information of PCR primers used in this study.
**Table S3**. Genes expression profiling of ‘*Candidatus* Liberibacter asiaticus’ in citrus and its parasitized dodder.
