# Supplementary material for: Establishment of a Cuscuta campestris‐mediated enrichment system for genomic and transcriptomic analyses of ‘Candidatus Liberibacter asiaticus’
Source: Microb Biotechnol. 2021 Mar 3;14(2):737–51. doi: 10.1111/1751-7915.13773 (PMC7936317; doi:10.1111/1751-7915.13773)
Supplement: Supplementary file 1 — Fig. S1. Evaluation of the genomic variation of ‘Candidatus Liberibacter asiaticus’ by the quality‐based variation detection based on reads mapping from citrus samples and its parasitized dodder samples. Reads mapping was generated by using dodder‐origin CLas genome (including the chromosomal region, strain‐C and prophage region, P‐strain‐1, 2, or 3) as reference for mapping with citrus HiSeq data. The quality‐based variation detection of each read mapping was performed with CLC Genomic workbench v9.5. Table S1. Quantification of ‘Candidatus Liberibacter asiaticus’ in citrus and the parasitized dodder and taxa‐specific PCR result. Table S2. General information of PCR primers used in this study. Table S3. Genes expression profiling of ‘Candidatus Liberibacter asiaticus’ in citrus and its parasitized dodder. [file MBT2-14-737-s001.docx]

**Fig. S1. Evaluation of the genomic variation of “*Candidatus* Liberibacter asiaticus” by the quality-based variation detection of reads mapping from citrus samples and its parasitized dodder samples.** Reads mapping was generated by using dodder-origin CLas genome (including the chromosomal region, strain-C and prophage region, P-strain-1, 2, or 3) as reference for mapping with citrus HiSeq data. The quality-based variation detection of each read mapping was performed with CLC Genomic workbench v9.5.


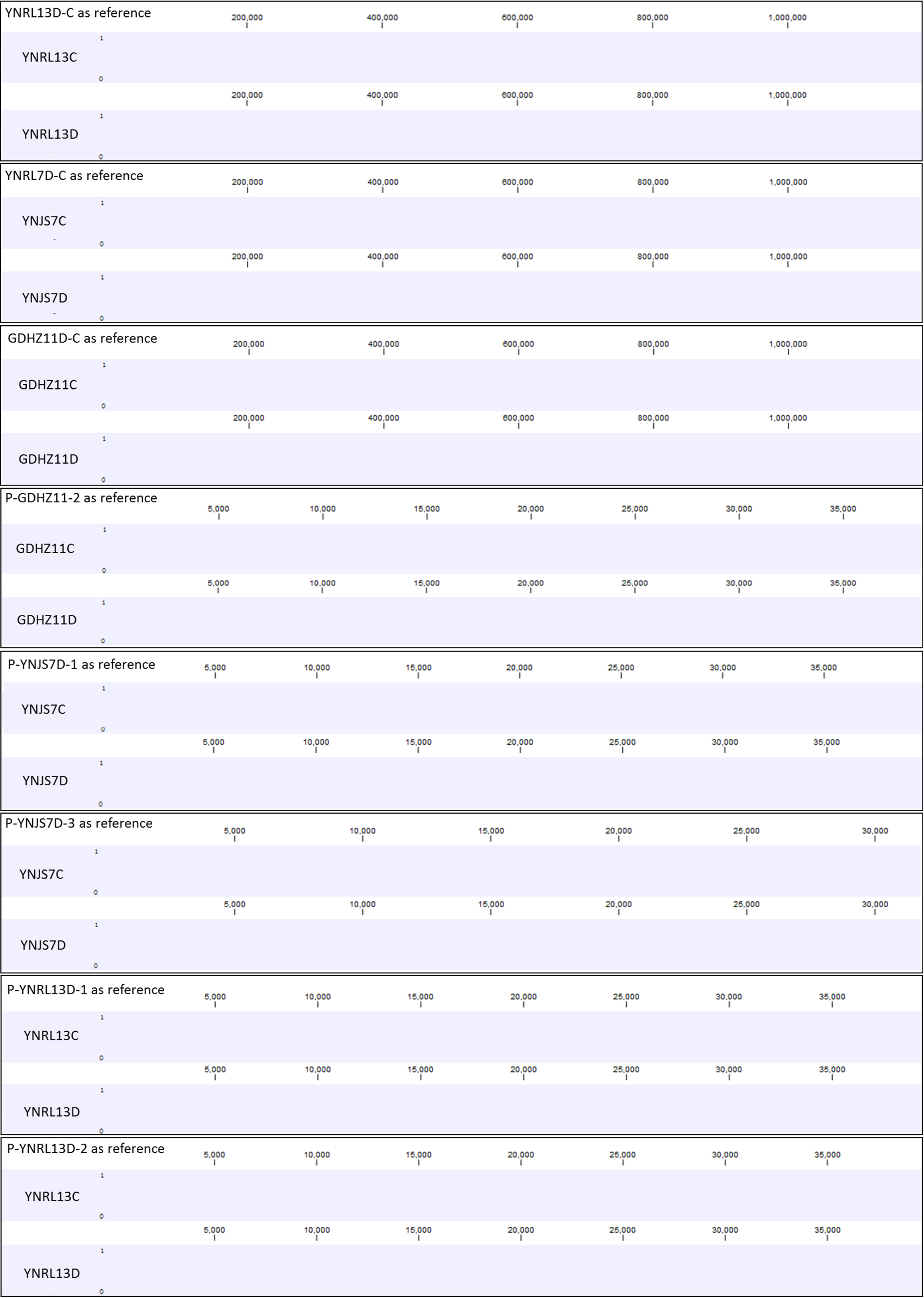


**Table S1. Quantification of “*Candidatus* Liberibacter asiaticus” in citrus and the parasitic dodder and taxa-specific PCR result.**

| No. | Set ID | Location | Variety | CLas cells / ng of total DNA | | Enrichment fold | Parasitized period | Type of appearance | “*Candidatus* Portiera aleyrodidarum” | | Citrus chlorotic dwarf associated virus | |
| --- | --- | --- | --- | --- | --- | --- | --- | --- | --- | --- | --- | --- |
|  |  |  |  | Citrus | Dodder |  |  |  | Citrus | Dodder | Citrus | Dodder |
| CLas-enriched group | | | | | | | | | | | | |
| 1 | TSZ-2-1 | Guangdong | "Shatangju" | 1,955 | 109,600 | 56.05 | 24 | IV | **30.52** | NF | NF | NF |
| 2 | TSZ-4-1 | Guangdong | "Shatangju" | 2,389 | 31,678 | 13.26 | 23 | III | **32.58** | NF | NF | NF |
| 3 | TSZ-5-1 | Guangdong | "Shatangju" | 815 | 11,928 | 14.63 | 24 | IV | **29.21** | NF | NF | NF |
| 4 | TSZ-6-3 | Guangdong | "Shatangju" | 5,482 | 193,109 | 35.23 | 21 | III | **27.82** | NF | NF | NF |
| 5 | C9-4 | Guangdong | "Shatangju" | 889 | 72,140 | 81.11 | 23 | IV | NA | NF | NF | NF |
| 6 | KS2-1-2 | Guangdong | "Shatangju" | 1,680 | 73,514 | 43.75 | 24 | III | NA | NF | NF | NF |
| 7 | C9-2 | Guangdong | "Shatangju" | 1,483 | 6,089 | 4.10 | 8 | II | **24.26** | NF | NF | NF |
| 8 | TSZ-7-2 | Guangdong | "Shatangju" | 1,796 | 3,170 | 1.76 | 12 | II | - | - | - | - |
| 9 | TSZ-22 | Guangdong | "Shatangju" | 1,903 | 52027 | 27.33 | 13 | IV | NF | NF | NF | NF |
| 10 | HZ-11 | Guangdong | "Shatangju" | 33,815 | 683,739 | 20.22 | 14 | III | - | - | - | - |
| 11 | YC-1 | Guangdong | "Shatangju" | 2,270 | 5,505 | 2.43 | 12 | II | NF | NF | NF | NF |
| 12 | T1-A1 | Guangdong | "Nianju" | 5,500 | 519,488 | 94.45 | 16 | IV | NF | NF | NF | NF |
| 13 | T1-A2 | Guangdong | "Nianju" | 5,645 | 314,898 | 55.78 | 16 | III | - | - | - | - |
| 14 | T1-A3 | Guangdong | "Nianju" | 2,486 | 214,080 | 86.11 | 16 | III | - | - | - | - |
| 15 | T1-A4 | Guangdong | "Nianju" | 2,702 | 94,541 | 35.00 | 16 | III | NF | NF | NF | NF |
| 16 | T1-A5 | Guangdong | "Nianju" | 2,815 | 291,598 | 103.58 | 16 | IV | NF | NF | NF | NF |
| 17 | T1-A6 | Guangdong | "Nianju" | 2,489 | 331,132 | 133.05 | 16 | IV | NF | NF | NF | NF |
| 18 | T1-A7 | Guangdong | "Nianju" | 1,291 | 541,089 | 418.97 | 17 | III | NF | NF | NF | NF |
| 19 | T1-A8 | Guangdong | "Nianju" | 1,684 | 99,338 | 58.98 | 17 | IV | NF | NF | NF | NF |
| 20 | RLi-2 | Yunnan | Lemon | 588 | 27,004 | 45.96 | 17 | III | NF | NF | **13.46** | **22.25** |
| 21 | RLi-3 | Yunnan | Lemon | 615 | 3,061 | 4.98 | 17 | II | **29.59** | NF | **13.42** | **23.23** |
| 22 | RLi-6 | Yunnan | Lemon | 485 | 5,936 | 12.25 | 17 | III | NF | NF | **26.51** | **29.95** |
| 23 | RLi-10 | Yunnan | Lemon | 971 | 7,537 | 7.76 | 12 | II | NF | NF | **12.56** | **22.76** |
| 24 | RLi-11 | Yunnan | Lemon | 292 | 491 | 1.68 | 10 | IV | NF | NF | **14.76** | **28.36** |
| 25 | RLi-13 | Yunnan | Lemon | 11,218 | 834,072 | 74.35 | 17 | III | - | - | - | - |
| 26 | JS-7 | Yunnan | Lemon | 15,911 | 144,690 | 9.09 | 17 | III | - | - | - | - |
| 27 | JS-6 | Yunnan | Lemon | 765 | 3,238 | 4.23 | 17 | II | NF | NF | **26.25** | **30.37** |
| 28 | Lia14 | Guangdong | "Gongkan" | 7,984 | 22,013 | 2.76 | 11 | II | - | - | - | - |
| 29 | Lin9 | Guangdong | "Gongkan" | 2,503 | 153,100 | 61.17 | 15 | IV | - | - | - | - |
| 30 | Lin12 | Guangdong | "Gongkan" | 2,226 | 203,020 | 91.20 | 15 | IV | - | - | - | - |
| (Ave.) |  |  |  | 4,088 | 168,428 | 53.37 | 16 |  |  |  |  |  |
| CLas non-enriched group | | | | | | | | | | | | |
| 1 | TSZ-2-3 | Guangdong | "Shatangju" | 1,594 | 215 | 0.14 | 5 | III | - | - | - | - |
| 2 | TSZ-6-2 | Guangdong | "Shatangju" | 1,577 | 86 | 0.05 | 8 | I | - | - | - | - |
| 3 | TSZ-8-1 | Guangdong | "Shatangju" | 1,595 | 408 | 0.26 | 8 | III | - | - | - | - |
| 4 | TSZ-7-4 | Guangdong | "Shatangju" | 827 | 294 | 0.36 | 8 | II | - | - | - | - |
| 5 | KS-2-1 | Guangdong | "Shatangju" | 7,827 | 1,624 | 0.21 | 6 | II | - | - | - | - |
| 6 | TSZ-8-4 | Guangdong | "Shatangju" | 1,621 | 120 | 0.07 | 8 | II | - | - | - | - |
| 7 | T1-1 | Guangdong | "Nianju" | 590 | 400 | 0.68 | 6 | II | - | - | - | - |
| 8 | T3-2 | Guangdong | "Nianju" | 3,324 | 971 | 0.29 | 8 | II | - | - | - | - |
| 9 | RL-7 | Yunnan | Lemon | 111 | 42 | 0.38 | 8 | I | NF | NF | **13.04** | **23.58** |
| 10 | RL-9 | Yunnan | Lemon | 364 | 135 | 0.37 | 5 | II | NF | NF | **26.44** | **29.86** |
| 11 | RL-12 | Yunnan | Lemon | 410 | 215 | 0.52 | 8 | I | NF | NF | **13.84** | **25.56** |
| 12 | JS-5 | Yunnan | Lemon | 3,523 | 500 | 0.14 | 5 | II | NF | NF | **26.90** | **30.31** |
| 13 | K4 | Guangdong | "Gongkan" | 3,392 | 565 | 0.17 | 5 | II | - | - | - | - |
| 14 | Lia6 | Guangdong | "Gongkan" | 2,110 | 216 | 0.10 | 8 | II | - | - | - | - |
| 15 | Lia2 | Guangdong | "Gongkan" | 7,065 | 2,500 | 0.35 | 8 | II | - | - | - | - |
| 16 | CH-1 | Guangdong | "Gongkan" | 6,692 | 4,179 | 0.62 | 5 | II | - | - | - | - |
| 17 | CH-2 | Guangdong | "Gongkan" | 5,360 | 1,291 | 0.24 | 8 | II | NF | NF | NF | NF |
| 18 | CH-14 | Guangdong | "Gongkan" | 3,075 | 144 | 0.05 | 8 | II | **30.22** | NF | NF | NF |
| (Ave.) |  |  |  | 2,836 | 772 | 0.27 | 6.5 |  | - | - | - | - |

**Table S2. General information of PCR primers used in this study.**

| Name | Sequence (5’ → 3’) | Amplicon size | Target gene / Microbe | Reference |
| --- | --- | --- | --- | --- |
| CLas-4G | AGTCGAGCGCGTATGCGAAT | 78 bp | 16S rRNA gene / "*Candidatus* Liberibacter asiaticus" (CLas) | Bao et al., 2019 |
| HLBr | GCGTTATCCCGTAGAAAAAGGTAG |  |  |  |
| HLBp | FAM-AGACGGGTGAGTAACGCG-BHQ |  |  |  |
| Por-3F | ACTTACTGCAGCTGAAGGATT | 80 bp | Glutamate-tRNA ligase (AGI27075.1) / "*Candidatus* Portiera aleyrodidarum" | This study |
| Por-3R | CTCCACCCTCTATACCAAAACG |  |  |  |
| CCDaV-2F | GGTGGCCGAGAAAGGAAAAG | 89 bp | Coat protein gene (YP_006522419.1) / Citrus chlorotic dwarf associated virus | This study |
| CCDaV-2R | AGGAACACCTTTGCTCGACT |  |  |  |
| rnpB-F | CATGAAAACTCAACGCCGGA | 114bp | Rnase P (CD16_05550) / CLas | This study |
| rnpB-R | GTGAGCTCTTACCCCACCAT |  |  |  |
| CD16_RS05425-F | GGAGGTTGTGATCTTTCTGACA | 93bp | Collagen-like protein (CD16_RS05425) / CLas | This study |
| CD16_RS05425-R | AGCCTTTTCTCTAGTGTTTTCCA |  |  |  |
| CD16_RS03055-F | TGGTGGTCGTGGAGTAGTTC | 88bp | Bacteria cell surface antigens (CD16_RS03055) / CLas | This study |
| CD16_RS03055-R | ACTCAGACCTGCATACGGAC |  |  |  |
| CD16_RS02370-F | TGTAGCTATTATTGCCGCCG | 80bp | Flp family type IVb pilin (CD16_RS02370) / CLas | This study |
| CD16_RS02370-R | TAGATATCCGGTTTGCGGCT |  |  |  |
| CD16_RS05110-F | CGTAAAACCACACCAGCTGT | 113bp | Hypothetical protein (CD16_RS05110) / CLas | This study |
| CD16_RS05110-R | GCTAGCATGCTCTCGATTCG |  |  |  |
| CD16_RS03700-F | GCTTTACAGTCTGCGGGTTC | 102bp | Chaperonin GroEL (CD16_RS03700) / CLas | This study |
| CD16_RS03700-R | GGCAGGCATTTGAGGAGATG |  |  |  |
| CD16_RS00895-F | GCAAGCAGTAACGCAACCTA | 85bp | Lytic transglycosylase (CD16_RS00895) / CLas | This study |
| CD16_RS00895-R | AGCCGATTTCCACTCTCCAA |  |  |  |
| CD16_RS03695-F | TGTACGCCGTCTTCAAAGTG | 113bp | Co-chaperone GroES (CD16_RS03695) / CLas | This study |
| CD16_RS03695-R | CAGCACCAACCCACATGATT |  |  |  |
| CD16_RS02830-F | AGTCCTGCTATACGACCTGC | 91bp | Molecular chaperone DnaK (CD16_RS02830) / CLas | This study |
| CD16_RS02830-R | AAACCGTTAGCAAAGCCGTT |  |  |  |
| CD16_RS02375-F | TGTAGCTATTATTGCCGCCG | 83bp | Flp family type IVb pilin (CD16_RS02375) / CLas | This study |
| CD16_RS02375-R | GGTGAGACATCTGGTTTGCG |  |  |  |
| CD16_RS00915-F | GCGCAATTATCGGAAAACCC | 80bp | Redoxin family protein (CD16_RS00915) / CLas | This study |
| CD16_RS00915-R | TCTTGGCGTTATGGAGCTCT |  |  |  |
| CD16_RS00940-F | GCCCTAACATCCGATTCAGC | 84bp | Redoxin family protein (CD16_RS00940) / CLas | This study |
| CD16_RS00940-R | AGGGGATGTTACAGGTGTCG |  |  |  |
| CD16_RS04750-F | AAATCGCTTGGTCGCAGTAG | 119bp | Glycoside hydrolase family protein (CD16_RS04750) / CLas | This study |
| CD16_RS04750-R | TACATTCCTCAGCGGCTTTT |  |  |  |
| CD16_RS01425-F | GCGTCGGATGCTTTCAAAGA | 111bp | Accessory factor UbiK family protein (CD16_RS01425) / CLas | This study |
| CD16_RS01425-R | GTTCTCGATCTCTTCTGCGC |  |  |  |
| CD16_RS02155-F | GTCCTCATCACCTCCCGATC | 105bp | Di-trans,poly-cis-decaprenylcis transferase (CD16_RS02155) / CLas | This study |
| CD16_RS02155-R | TCTATGCCCTTACGGTGTCC |  |  |  |
| CD16_RS03415-F | GATCTGCTGTGCCCAAAGG | 107bp | Flagellar hook-associated protein FlgK (CD16_RS03415) / CLas | This study |
| CD16_RS03415-R | ACCAATAAGAACCCTCGCCA |  |  |  |
| CD16_RS01050-F | CGCTTAGCACTCGTTACAGG | 99bp | SDR family NAD(P)-dependent oxidoreductase (CD16_RS01050) / CLas | This study |
| CD16_RS01050-R | ACTACGACCACAAGCGATGA |  |  |  |
| CD16_RS04575-F | ACCAACAAGAACAAGTCCTGC | 119bp | Glycoside hydrolase family protein (CD16_RS04750) / CLas | This study |
| CD16_RS04575-R | AGTCGTCCCAGAAACTGTCG |  |  |  |
| CD16_RS03380-F | CAGGATCGCGTTTCTTCAGG | 110bp | Flagellin (CD16_RS03380) / CLas | This study |
| CD16_RS03380-R | GAAACTGCAGAAAGAGCCCC |  |  |  |
| CD16_RS03045-F | GCCGCCCATATTGAGTACCT | 102bp | 3-phosphatidyl transferase (CD16_RS03045) / CLas | This study |
| CD16_RS03045-R | AGGTCGCATGCTTGATCCTA |  |  |  |
| CD16_RS01875-F | ACCCAACAGCTAGCACTCAT | 105bp | 16S ribosomal RNA (CD16_RS01875) / CLas | This study |
| CD16_RS01875-R | GCTCACTGGCCTGATACTGA |  |  |  |

**Table S3. Genes expression profiling of “*Candidatus* Liberibacter asiaticus” in citrus and its parasitic dodder.**

| No. | Gene locus | Citrus^a^ | | Dodder | |
| --- | --- | --- | --- | --- | --- |
|  |  | TPM | Total gene reads | TPM | Total gene reads |
| 1 | CD16_RS00015 | 0 | 0 | 0 | 0 |
| 2 | CD16_RS00030 | 0 | 0 | 0 | 0 |
| 3 | CD16_RS00100 | 0 | 0 | 0 | 0 |
| 4 | CD16_RS00110 | 0 | 0 | 0 | 0 |
| 5 | rpmJ | 0 | 0 | 0 | 0 |
| 6 | CD16_RS00230 | 0 | 0 | 0 | 0 |
| 7 | CD16_RS00240 | 0 | 0 | 0 | 0 |
| 8 | CD16_RS00250 | 0 | 0 | 0 | 0 |
| 9 | CD16_RS00315 | 0 | 0 | 0 | 0 |
| 10 | CD16_RS00350 | 0 | 0 | 0 | 0 |
| 11 | CD16_RS00390 | 0 | 0 | 0 | 0 |
| 12 | CD16_RS00735 | 0 | 0 | 0 | 0 |
| 13 | CD16_RS00910 | 0 | 0 | 0 | 0 |
| 14 | CD16_RS00925 | 0 | 0 | 0 | 0 |
| 15 | CD16_RS00935 | 0 | 0 | 0 | 0 |
| 16 | CD16_RS01065 | 0 | 0 | 0 | 0 |
| 17 | CD16_RS01290 | 0 | 0 | 0 | 0 |
| 18 | CD16_RS01430 | 0 | 0 | 0 | 0 |
| 19 | CD16_RS01645 | 0 | 0 | 0 | 0 |
| 20 | CD16_RS01670 | 0 | 0 | 0 | 0 |
| 21 | CD16_RS01725 | 0 | 0 | 0 | 0 |
| 22 | rpmH | 0 | 0 | 0 | 0 |
| 23 | CD16_RS01850 | 0 | 0 | 0 | 0 |
| 24 | rrf_1 | 0 | 0 | 0 | 0 |
| 25 | CD16_RS01865 | 0 | 0 | 0 | 0 |
| 26 | CD16_RS01870 | 0 | 0 | 0 | 0 |
| 27 | ffs | 0 | 0 | 0 | 0 |
| 28 | CD16_RS02260 | 0 | 0 | 0 | 0 |
| 29 | CD16_RS02510 | 0 | 0 | 0 | 0 |
| 30 | CD16_RS02725 | 0 | 0 | 0 | 0 |
| 31 | CD16_RS02770 | 0 | 0 | 0 | 0 |
| 32 | CD16_RS02795 | 0 | 0 | 0 | 0 |
| 33 | CD16_RS02840 | 0 | 0 | 0 | 0 |
| 34 | CD16_RS02960 | 0 | 0 | 0 | 0 |
| 35 | CD16_RS03020 | 0 | 0 | 0 | 0 |
| 36 | CD16_RS03025 | 0 | 0 | 0 | 0 |
| 37 | CD16_RS05640 | 0 | 0 | 0 | 0 |
| 38 | CD16_RS03260 | 0 | 0 | 0 | 0 |
| 39 | CD16_RS03375 | 0 | 0 | 0 | 0 |
| 40 | CD16_RS03445 | 0 | 0 | 0 | 0 |
| 41 | CD16_RS03565 | 0 | 0 | 0 | 0 |
| 42 | CD16_RS03580 | 0 | 0 | 0 | 0 |
| 43 | CD16_RS03585 | 0 | 0 | 0 | 0 |
| 44 | rrf_2 | 0 | 0 | 0 | 0 |
| 45 | CD16_RS03600 | 0 | 0 | 0 | 0 |
| 46 | CD16_RS03650 | 0 | 0 | 0 | 0 |
| 47 | CD16_RS03670 | 0 | 0 | 0 | 0 |
| 48 | CD16_RS03705 | 0 | 0 | 0 | 0 |
| 49 | CD16_RS03710 | 0 | 0 | 0 | 0 |
| 50 | CD16_RS03715 | 0 | 0 | 0 | 0 |
| 51 | CD16_RS03855 | 0 | 0 | 0 | 0 |
| 52 | CD16_RS03860 | 0 | 0 | 0 | 0 |
| 53 | rrf_3 | 0 | 0 | 0 | 0 |
| 54 | CD16_RS03875 | 0 | 0 | 0 | 0 |
| 55 | CD16_RS04030 | 0 | 0 | 0 | 0 |
| 56 | CD16_RS04100 | 0 | 0 | 0 | 0 |
| 57 | CD16_RS04130 | 0 | 0 | 0 | 0 |
| 58 | CD16_RS04350 | 0 | 0 | 0 | 0 |
| 59 | CD16_RS04380 | 0 | 0 | 0 | 0 |
| 60 | CD16_RS04475 | 0 | 0 | 0 | 0 |
| 61 | CD16_RS04510 | 0 | 0 | 0 | 0 |
| 62 | CD16_RS04520 | 0 | 0 | 0 | 0 |
| 63 | CD16_RS04820 | 0 | 0 | 0 | 0 |
| 64 | CD16_RS04830 | 0 | 0 | 0 | 0 |
| 65 | CD16_RS04945 | 0 | 0 | 0 | 0 |
| 66 | CD16_RS05675 | 0 | 0 | 0 | 0 |
| 67 | CD16_RS05265 | 0 | 0 | 0 | 0 |
| 68 | CD16_RS05360 | 0 | 0 | 0 | 0 |
| 69 | CD16_RS05410 | 0 | 0 | 26.3 | 1 |
| 70 | CD16_RS02820 | 0 | 0 | 28.43 | 1 |
| 71 | CD16_RS05115 | 0 | 0 | 30.35 | 1 |
| 72 | CD16_RS05145 | 0 | 0 | 37.57 | 1 |
| 73 | CD16_RS02860 | 0 | 0 | 43.84 | 1 |
| 74 | CD16_RS04925 | 0 | 0 | 43.84 | 1 |
| 75 | CD16_RS03200 | 0 | 0 | 44.45 | 1 |
| 76 | CD16_RS00140 | 0 | 0 | 45.74 | 1 |
| 77 | CD16_RS02810 | 0 | 0 | 47.11 | 1 |
| 78 | CD16_RS05100 | 0 | 0 | 52.6 | 1 |
| 79 | CD16_RS02390 | 0 | 0 | 53.5 | 1 |
| 80 | CD16_RS04125 | 0 | 0 | 55.37 | 1 |
| 81 | CD16_RS05670 | 0 | 0 | 57.39 | 1 |
| 82 | CD16_RS05400 | 0 | 0 | 63.76 | 2 |
| 83 | CD16_RS04920 | 0 | 0 | 77.93 | 2 |
| 84 | CD16_RS04015 | 0 | 0 | 78.91 | 4 |
| 85 | CD16_RS01710 | 0 | 0 | 80.47 | 3 |
| 86 | CD16_RS05540 | 0 | 0 | 91.49 | 2 |
| 87 | CD16_RS03450 | 0 | 0 | 92.83 | 3 |
| 88 | flgC | 0 | 0 | 93.52 | 4 |
| 89 | CD16_RS01950 | 0 | 0 | 98.63 | 2 |
| 90 | CD16_RS04485 | 0 | 0 | 100.73 | 3 |
| 91 | CD16_RS02800 | 0 | 0 | 102.92 | 3 |
| 92 | fliQ | 0 | 0 | 106.39 | 3 |
| 93 | CD16_RS05070 | 0 | 0 | 110.1 | 3 |
| 94 | CD16_RS05185 | 0 | 0 | 122.97 | 3 |
| 95 | CD16_RS04400 | 0 | 0 | 132.9 | 4 |
| 96 | CD16_RS05380 | 0 | 0 | 134.88 | 10 |
| 97 | CD16_RS05570 | 0 | 0 | 137.23 | 3 |
| 98 | CD16_RS05020 | 0 | 0 | 145.12 | 4 |
| 99 | CD16_RS05345 | 0 | 0 | 146.8 | 4 |
| 100 | ruvC | 0 | 0 | 148.53 | 8 |
| 101 | CD16_RS05610 | 0 | 0 | 151.74 | 5 |
| 102 | CD16_RS00130 | 0 | 0 | 152.11 | 4 |
| 103 | CD16_RS00855 | 0 | 0 | 159.14 | 6 |
| 104 | CD16_RS00880 | 0 | 0 | 160.29 | 14 |
| 105 | CD16_RS05620 | 0 | 0 | 164.39 | 5 |
| 106 | CD16_RS02385 | 0 | 0 | 166.12 | 3 |
| 107 | CD16_RS03490 | 0 | 0 | 167.59 | 6 |
| 108 | CD16_RS00345 | 0 | 0 | 180.36 | 4 |
| 109 | CD16_RS04430 | 0 | 0 | 188.43 | 4 |
| 110 | CD16_RS04405 | 0 | 0 | 190.9 | 15 |
| 111 | CD16_RS02815 | 0 | 0 | 206.97 | 8 |
| 112 | CD16_RS03255 | 0 | 0 | 208.68 | 8 |
| 113 | CD16_RS05340 | 0 | 0 | 219.19 | 5 |
| 114 | CD16_RS03470 | 0 | 0 | 235.54 | 5 |
| 115 | CD16_RS05390 | 0 | 0 | 237.91 | 15 |
| 116 | CD16_RS02870 | 0 | 0 | 239.11 | 5 |
| 117 | CD16_RS00310 | 0 | 0 | 241.19 | 27 |
| 118 | rpmD | 0 | 0 | 242.79 | 5 |
| 119 | CD16_RS00010 | 0 | 0 | 253.18 | 15 |
| 120 | CD16_RS05545 | 0 | 0 | 272.76 | 7 |
| 121 | CD16_RS02360 | 0 | 0 | 291.35 | 6 |
| 122 | CD16_RS00905 | 0 | 0 | 315.63 | 12 |
| 123 | CD16_RS04805 | 0 | 0 | 318.82 | 10 |
| 124 | CD16_RS01240 | 0 | 0 | 340.73 | 19 |
| 125 | CD16_RS04225 | 0 | 0 | 356.35 | 28 |
| 126 | queE | 0 | 0 | 372.2 | 25 |
| 127 | CD16_RS05125 | 0 | 0 | 376.24 | 18 |
| 128 | secE | 0 | 0 | 417.74 | 9 |
| 129 | CD16_RS02015 | 0 | 0 | 475.34 | 25 |
| 130 | CD16_RS02995 | 0 | 0 | 502.13 | 21 |
| 131 | smpB | 0 | 0 | 512.89 | 26 |
| 132 | erpA | 0 | 0 | 545.17 | 19 |
| 133 | CD16_RS01715 | 0 | 0 | 906.97 | 25 |
| 134 | CD16_RS00025 | 20.01 | 1 | 246.95 | 32 |
| 135 | CD16_RS05365 | 22.24 | 1 | 25.73 | 3 |
| 136 | CD16_RS05035 | 29.77 | 1 | 218.07 | 19 |
| 137 | fliR | 32.87 | 1 | 240.84 | 19 |
| 138 | CD16_RS02265 | 37.72 | 1 | 479.99 | 33 |
| 139 | CD16_RS00755 | 38.43 | 1 | 385.27 | 26 |
| 140 | CD16_RS02740 | 41.27 | 1 | 350.11 | 22 |
| 141 | CD16_RS01780 | 45.86 | 2 | 274.07 | 31 |
| 142 | CD16_RS00020 | 46.91 | 2 | 217.05 | 24 |
| 143 | CD16_RS03390 | 47.59 | 2 | 192.68 | 21 |
| 144 | CD16_RS05395 | 48.44 | 4 | 126.06 | 27 |
| 145 | CD16_RS03150 | 56.85 | 2 | 504.13 | 46 |
| 146 | CD16_RS04675 | 56.85 | 1 | 526.05 | 24 |
| 147 | rsmA | 57.44 | 2 | 398.69 | 36 |
| 148 | CD16_RS01285 | 58.47 | 1 | 383.26 | 17 |
| 149 | CD16_RS03485 | 59.32 | 1 | 45.74 | 2 |
| 150 | CD16_RS04415 | 59.32 | 1 | 160.1 | 7 |
| 151 | CD16_RS04515 | 60.04 | 3 | 239.23 | 31 |
| 152 | flgB | 62.49 | 1 | 168.66 | 7 |
| 153 | truA | 66.01 | 2 | 254.54 | 20 |
| 154 | mvaD | 68.98 | 3 | 212.78 | 24 |
| 155 | CD16_RS02045 | 70.57 | 2 | 95.23 | 7 |
| 156 | CD16_RS04195 | 71.49 | 2 | 234.31 | 17 |
| 157 | CD16_RS05615 | 72.76 | 2 | 126.25 | 9 |
| 158 | CD16_RS04855 | 73.09 | 1 | 140.91 | 5 |
| 159 | CD16_RS04570 | 75.56 | 3 | 242.79 | 25 |
| 160 | CD16_RS00125 | 78.33 | 2 | 422.85 | 28 |
| 161 | nuoK | 79.47 | 1 | 459.65 | 15 |
| 162 | CD16_RS05095 | 80.25 | 1 | 123.78 | 4 |
| 163 | CD16_RS01845 | 80.25 | 1 | 371.33 | 12 |
| 164 | recR | 81.05 | 2 | 578.13 | 37 |
| 165 | CD16_RS00465 | 83.53 | 1 | 289.86 | 9 |
| 166 | CD16_RS04005 | 84.39 | 1 | 130.16 | 4 |
| 167 | CD16_RS05350 | 86.17 | 1 | 365.46 | 11 |
| 168 | atpH | 87.55 | 2 | 607.62 | 36 |
| 169 | CD16_RS01245 | 88.49 | 4 | 230.32 | 27 |
| 170 | CD16_RS01060 | 91.97 | 1 | 70.93 | 2 |
| 171 | CD16_RS02275 | 92.67 | 3 | 190.57 | 16 |
| 172 | CD16_RS05105 | 95.18 | 1 | 440.41 | 12 |
| 173 | CD16_RS02560 | 97.16 | 4 | 393.36 | 42 |
| 174 | CD16_RS03575 | 98.1 | 6 | 12.61 | 2 |
| 175 | CD16_RS05180 | 98.62 | 1 | 38.03 | 1 |
| 176 | CD16_RS05215 | 98.62 | 2 | 76.05 | 4 |
| 177 | CD16_RS05385 | 98.62 | 2 | 209.15 | 11 |
| 178 | CD16_RS03885 | 99.72 | 4 | 221.1 | 23 |
| 179 | phrB | 101.48 | 6 | 247.81 | 38 |
| 180 | CD16_RS05135 | 102.32 | 2 | 650.98 | 33 |
| 181 | CD16_RS04460 | 103.62 | 1 | 119.86 | 3 |
| 182 | CD16_RS03560 | 103.95 | 4 | 260.52 | 26 |
| 183 | CD16_RS01085 | 104.94 | 4 | 647.44 | 64 |
| 184 | CD16_RS05165 | 106.31 | 1 | 245.94 | 6 |
| 185 | CD16_RS04930 | 106.58 | 5 | 65.76 | 8 |
| 186 | flgA | 107 | 2 | 330.07 | 16 |
| 187 | CD16_RS03810 | 107.24 | 3 | 385.92 | 28 |
| 188 | CD16_RS05120 | 107.99 | 5 | 283.15 | 34 |
| 189 | tgt | 107.99 | 5 | 366.43 | 44 |
| 190 | CD16_RS03405 | 109.63 | 3 | 267.72 | 19 |
| 191 | alr | 110.32 | 5 | 170.15 | 20 |
| 192 | fliN | 110.62 | 2 | 149.28 | 7 |
| 193 | CD16_RS01370 | 113.69 | 1 | 394.53 | 9 |
| 194 | CD16_RS03925 | 113.69 | 3 | 789.07 | 54 |
| 195 | CD16_RS00990 | 115.29 | 2 | 355.64 | 16 |
| 196 | CD16_RS00155 | 115.29 | 5 | 453.44 | 51 |
| 197 | kdsA | 116.11 | 4 | 246.23 | 22 |
| 198 | CD16_RS04470 | 116.32 | 8 | 313.95 | 56 |
| 199 | CD16_RS03455 | 116.94 | 1 | 180.36 | 4 |
| 200 | CD16_RS05240 | 120.38 | 1 | 278.49 | 6 |
| 201 | rpmI | 120.38 | 1 | 1,113.98 | 24 |
| 202 | CD16_RS05605 | 121.09 | 10 | 359.52 | 77 |
| 203 | pdxH | 121.57 | 3 | 359.38 | 23 |
| 204 | CD16_RS04865 | 122.63 | 4 | 461.03 | 39 |
| 205 | CD16_RS04580 | 122.99 | 6 | 173.88 | 22 |
| 206 | CD16_RS02250 | 123.28 | 5 | 589.42 | 62 |
| 207 | CD16_RS05220 | 124.03 | 1 | 143.47 | 3 |
| 208 | CD16_RS04655 | 124.03 | 1 | 191.29 | 4 |
| 209 | flgG | 124.5 | 4 | 168.01 | 14 |
| 210 | CD16_RS05565 | 125.93 | 2 | 485.58 | 20 |
| 211 | CD16_RS00430 | 127.9 | 1 | 838.38 | 17 |
| 212 | CD16_RS01875 | 130.8 | 8 | 1,733.64 | 275 |
| 213 | CD16_RS05370 | 132.03 | 3 | 118.78 | 7 |
| 214 | CD16_RS02580 | 132.56 | 4 | 127.78 | 10 |
| 215 | CD16_RS01045 | 133.92 | 8 | 458.27 | 71 |
| 216 | CD16_RS01145 | 135.3 | 2 | 130.42 | 5 |
| 217 | CD16_RS00005 | 136.43 | 2 | 157.81 | 6 |
| 218 | CD16_RS00900 | 136.43 | 1 | 157.81 | 3 |
| 219 | hemJ | 137.19 | 3 | 211.59 | 12 |
| 220 | CD16_RS03500 | 138.74 | 3 | 160.49 | 9 |
| 221 | CD16_RS05190 | 139.93 | 6 | 251.78 | 28 |
| 222 | CD16_RS04115 | 140.1 | 7 | 208.36 | 27 |
| 223 | CD16_RS05150 | 142.36 | 2 | 54.89 | 2 |
| 224 | CD16_RS05140 | 144.03 | 6 | 222.14 | 24 |
| 225 | CD16_RS03395 | 144.33 | 7 | 333.91 | 42 |
| 226 | CD16_RS01375 | 144.88 | 6 | 223.45 | 24 |
| 227 | CD16_RS00380 | 145.06 | 7 | 447.47 | 56 |
| 228 | CD16_RS03465 | 147.49 | 2 | 142.17 | 5 |
| 229 | CD16_RS00120 | 147.49 | 2 | 454.96 | 16 |
| 230 | yajC | 148.83 | 2 | 401.71 | 14 |
| 231 | lpxB | 149.22 | 7 | 361.66 | 44 |
| 232 | CD16_RS01260 | 150.2 | 2 | 173.74 | 6 |
| 233 | mfd | 151.59 | 22 | 313.5 | 118 |
| 234 | CD16_RS03615 | 154.03 | 7 | 246.05 | 29 |
| 235 | CD16_RS03920 | 155.92 | 2 | 150.3 | 5 |
| 236 | CD16_RS05655 | 157.42 | 2 | 151.74 | 5 |
| 237 | tsaB | 158.18 | 4 | 213.47 | 14 |
| 238 | CD16_RS03430 | 159.46 | 3 | 184.46 | 9 |
| 239 | CD16_RS02595 | 161.41 | 7 | 293.4 | 33 |
| 240 | CD16_RS05375 | 161.77 | 5 | 261.98 | 21 |
| 241 | CD16_RS03850 | 163.5 | 10 | 18.91 | 3 |
| 242 | CD16_RS03400 | 164.54 | 8 | 182.4 | 23 |
| 243 | CD16_RS04205 | 165.37 | 2 | 350.7 | 11 |
| 244 | fliG | 165.61 | 7 | 200.69 | 22 |
| 245 | argB | 166.49 | 6 | 460.07 | 43 |
| 246 | CD16_RS02730 | 167.05 | 5 | 219.01 | 17 |
| 247 | CD16_RS00460 | 167.05 | 2 | 289.86 | 9 |
| 248 | motA | 168.78 | 6 | 141 | 13 |
| 249 | obgE | 170.54 | 7 | 356.96 | 38 |
| 250 | CD16_RS04145 | 174.63 | 8 | 336.67 | 40 |
| 251 | CD16_RS05050 | 176.67 | 9 | 325.47 | 43 |
| 252 | trxB | 177.95 | 7 | 519.51 | 53 |
| 253 | addA | 179.45 | 26 | 252.82 | 95 |
| 254 | CD16_RS05435 | 179.91 | 2 | 104.05 | 3 |
| 255 | CD16_RS05505 | 179.91 | 2 | 867.11 | 25 |
| 256 | CD16_RS04040 | 180.13 | 9 | 223.79 | 29 |
| 257 | CD16_RS05040 | 180.3 | 5 | 528.36 | 38 |
| 258 | CD16_RS05160 | 182.92 | 4 | 299.76 | 17 |
| 259 | CD16_RS02700 | 183.54 | 10 | 467.07 | 66 |
| 260 | CD16_RS04010 | 183.95 | 2 | 106.39 | 3 |
| 261 | CD16_RS03905 | 188.18 | 2 | 145.12 | 4 |
| 262 | CD16_RS00500 | 188.72 | 8 | 209.21 | 23 |
| 263 | flhA | 188.99 | 16 | 259.61 | 57 |
| 264 | ubiB | 189.63 | 12 | 316.85 | 52 |
| 265 | murF | 190.36 | 11 | 380.35 | 57 |
| 266 | CD16_RS02005 | 190.36 | 7 | 419.44 | 40 |
| 267 | CD16_RS00175 | 191 | 7 | 399.79 | 38 |
| 268 | gloB | 191.11 | 6 | 147.37 | 12 |
| 269 | CD16_RS02630 | 192.04 | 8 | 675.68 | 73 |
| 270 | purS | 192.6 | 2 | 594.12 | 16 |
| 271 | CD16_RS04450 | 194.9 | 3 | 200.4 | 8 |
| 272 | CD16_RS03175 | 194.9 | 11 | 396.24 | 58 |
| 273 | CD16_RS04620 | 195.36 | 10 | 308.85 | 41 |
| 274 | ruvB | 195.48 | 8 | 150.75 | 16 |
| 275 | CD16_RS00055 | 195.83 | 5 | 483.26 | 32 |
| 276 | recF | 195.93 | 9 | 436.51 | 52 |
| 277 | CD16_RS01900 | 196.13 | 19 | 374.14 | 94 |
| 278 | purE | 197.25 | 4 | 361.26 | 19 |
| 279 | CD16_RS02805 | 197.59 | 7 | 152.37 | 14 |
| 280 | CD16_RS03495 | 198.04 | 3 | 458.17 | 18 |
| 281 | CD16_RS05580 | 198.04 | 3 | 1,425.41 | 56 |
| 282 | CD16_RS03190 | 198.68 | 5 | 398.36 | 26 |
| 283 | CD16_RS05245 | 199.65 | 6 | 654.35 | 51 |
| 284 | ychF | 200.19 | 9 | 411.69 | 48 |
| 285 | CD16_RS02930 | 201.29 | 18 | 189.72 | 44 |
| 286 | CD16_RS02145 | 202.12 | 6 | 532.54 | 41 |
| 287 | fabZ | 202.12 | 4 | 545.53 | 28 |
| 288 | CD16_RS01540 | 202.95 | 9 | 634.73 | 73 |
| 289 | xth | 203.19 | 7 | 358.16 | 32 |
| 290 | pgsA | 203.62 | 5 | 109.92 | 7 |
| 291 | CD16_RS05015 | 205.15 | 10 | 435.08 | 55 |
| 292 | CD16_RS02610 | 205.28 | 8 | 375.98 | 38 |
| 293 | CD16_RS04780 | 206.36 | 3 | 132.62 | 5 |
| 294 | genX | 206.94 | 9 | 363.5 | 41 |
| 295 | CD16_RS05560 | 206.97 | 20 | 686.32 | 172 |
| 296 | CD16_RS01150 | 207.23 | 10 | 383.55 | 48 |
| 297 | CD16_RS02850 | 209.4 | 11 | 396.37 | 54 |
| 298 | recG | 210.19 | 18 | 243.14 | 54 |
| 299 | hemF | 212.62 | 8 | 430.4 | 42 |
| 300 | pdxA | 214.16 | 9 | 192.68 | 21 |
| 301 | ung | 215.41 | 6 | 221.49 | 16 |
| 302 | CD16_RS01450 | 215.41 | 3 | 498.36 | 18 |
| 303 | CD16_RS02000 | 215.41 | 7 | 664.48 | 56 |
| 304 | CD16_RS05475 | 215.41 | 3 | 885.97 | 32 |
| 305 | CD16_RS02225 | 216.84 | 4 | 627.07 | 30 |
| 306 | CD16_RS00535 | 218.28 | 2 | 547.09 | 13 |
| 307 | CD16_RS01560 | 218.7 | 7 | 301.17 | 25 |
| 308 | CD16_RS01050 | 222.09 | 7 | 110.1 | 9 |
| 309 | CD16_RS03785 | 222.16 | 13 | 474.43 | 72 |
| 310 | fliI | 223.75 | 12 | 323.54 | 45 |
| 311 | CD16_RS04490 | 225.29 | 3 | 1,013.48 | 35 |
| 312 | mutM | 225.81 | 8 | 272.09 | 25 |
| 313 | CD16_RS03385 | 226.33 | 6 | 189.09 | 13 |
| 314 | dnaE | 227.19 | 34 | 458.63 | 178 |
| 315 | CD16_RS00190 | 227.38 | 2 | 131.51 | 3 |
| 316 | CD16_RS02885 | 227.38 | 11 | 231.14 | 29 |
| 317 | CD16_RS04505 | 230.58 | 2 | 133.36 | 3 |
| 318 | CD16_RS01400 | 230.58 | 10 | 293.4 | 33 |
| 319 | CD16_RS02685 | 231.67 | 3 | 267.99 | 9 |
| 320 | CD16_RS03410 | 232.77 | 12 | 194.46 | 26 |
| 321 | CD16_RS05025 | 233.32 | 12 | 299.88 | 40 |
| 322 | CD16_RS01195 | 233.88 | 5 | 180.36 | 10 |
| 323 | yidD | 233.88 | 4 | 180.36 | 8 |
| 324 | CD16_RS04120 | 234.91 | 13 | 376.24 | 54 |
| 325 | CD16_RS03805 | 235 | 12 | 490.81 | 65 |
| 326 | CD16_RS04900 | 235.22 | 5 | 834.42 | 46 |
| 327 | CD16_RS02010 | 236.58 | 10 | 346.64 | 38 |
| 328 | lnt | 236.58 | 15 | 468.27 | 77 |
| 329 | CD16_RS01600 | 237.96 | 5 | 256.91 | 14 |
| 330 | rplX | 238.42 | 3 | 612.87 | 20 |
| 331 | CD16_RS02980 | 240.05 | 10 | 527.59 | 57 |
| 332 | CD16_RS01380 | 241.3 | 13 | 372.17 | 52 |
| 333 | CD16_RS01340 | 241.4 | 11 | 465.4 | 55 |
| 334 | murB | 241.47 | 10 | 186.21 | 20 |
| 335 | thiQ | 243.83 | 7 | 201.46 | 15 |
| 336 | CD16_RS00420 | 244.35 | 2 | 471.09 | 10 |
| 337 | recJ | 245.16 | 18 | 257.33 | 49 |
| 338 | CD16_RS00815 | 245.82 | 10 | 208.52 | 22 |
| 339 | CD16_RS05170 | 246.8 | 10 | 371.14 | 39 |
| 340 | CD16_RS05045 | 248.05 | 8 | 227.16 | 19 |
| 341 | CD16_RS01415 | 248.05 | 11 | 495.61 | 57 |
| 342 | CD16_RS04595 | 249.56 | 5 | 230.95 | 12 |
| 343 | CD16_RS05155 | 250.58 | 6 | 96.62 | 6 |
| 344 | CD16_RS01405 | 251.87 | 2 | 97.12 | 2 |
| 345 | CD16_RS03625 | 252.26 | 20 | 442.56 | 91 |
| 346 | CD16_RS02280 | 253.43 | 10 | 332.24 | 34 |
| 347 | CD16_RS00045 | 253.82 | 8 | 367.01 | 30 |
| 348 | pncB | 253.97 | 13 | 248.58 | 33 |
| 349 | ruvX | 254.21 | 5 | 215.65 | 11 |
| 350 | CD16_RS03250 | 254.21 | 5 | 411.69 | 21 |
| 351 | ftsY | 254.21 | 10 | 431.29 | 44 |
| 352 | rimP | 254.48 | 6 | 572.38 | 35 |
| 353 | rpe | 254.67 | 7 | 420.84 | 30 |
| 354 | CD16_RS04790 | 255.08 | 11 | 295.06 | 33 |
| 355 | pyrC | 257.26 | 11 | 541.08 | 60 |
| 356 | CD16_RS01350 | 257.41 | 10 | 625.3 | 63 |
| 357 | efp | 258.5 | 6 | 365.46 | 22 |
| 358 | thiP | 259.62 | 17 | 235.54 | 40 |
| 359 | bioD | 262.84 | 7 | 593.61 | 41 |
| 360 | queF | 264.05 | 5 | 366.53 | 18 |
| 361 | CD16_RS02775 | 264.05 | 7 | 450.9 | 31 |
| 362 | rpsS | 264.05 | 3 | 610.89 | 18 |
| 363 | CD16_RS05600 | 264.05 | 7 | 945.43 | 65 |
| 364 | hemC | 265.77 | 10 | 215.2 | 21 |
| 365 | CD16_RS01615 | 265.89 | 28 | 289.26 | 79 |
| 366 | CD16_RS05200 | 266.2 | 4 | 102.64 | 4 |
| 367 | CD16_RS04440 | 267.51 | 5 | 453.84 | 22 |
| 368 | murJ_1 | 268.12 | 17 | 541.25 | 89 |
| 369 | CD16_RS04375 | 268.38 | 4 | 413.94 | 16 |
| 370 | CD16_RS03005 | 268.38 | 20 | 450.16 | 87 |
| 371 | CD16_RS01805 | 269.01 | 7 | 533.45 | 36 |
| 372 | galE | 269.59 | 11 | 387.45 | 41 |
| 373 | tsaD | 269.86 | 12 | 251.46 | 29 |
| 374 | CD16_RS00170 | 271.95 | 10 | 629.16 | 60 |
| 375 | radA | 272.86 | 16 | 322.2 | 49 |
| 376 | CD16_RS01190 | 272.86 | 10 | 557.61 | 53 |
| 377 | CD16_RS01155 | 272.86 | 5 | 568.13 | 27 |
| 378 | CD16_RS03995 | 273.87 | 9 | 539.73 | 46 |
| 379 | flgH | 274 | 8 | 303.74 | 23 |
| 380 | CD16_RS02495 | 275.61 | 10 | 329.44 | 31 |
| 381 | CD16_RS04465 | 278.42 | 5 | 300.6 | 14 |
| 382 | CD16_RS00790 | 278.9 | 20 | 612.97 | 114 |
| 383 | CD16_RS03065 | 279.69 | 15 | 481.71 | 67 |
| 384 | CD16_RS04650 | 281.05 | 8 | 379.29 | 28 |
| 385 | rfbD | 281.29 | 10 | 347.08 | 32 |
| 386 | rpiA | 282.26 | 8 | 340.12 | 25 |
| 387 | CD16_RS01185 | 282.26 | 9 | 592.56 | 49 |
| 388 | CD16_RS03280 | 283.49 | 8 | 601.19 | 44 |
| 389 | CD16_RS02055 | 284.05 | 11 | 547.62 | 55 |
| 390 | CD16_RS05405 | 284.1 | 16 | 198.55 | 29 |
| 391 | CD16_RS01935 | 286.5 | 7 | 457.66 | 29 |
| 392 | CD16_RS00850 | 287.22 | 6 | 295.32 | 16 |
| 393 | CD16_RS01585 | 288.23 | 5 | 177.82 | 8 |
| 394 | CD16_RS01890 | 288.6 | 11 | 222.56 | 22 |
| 395 | CD16_RS00845 | 289.53 | 11 | 182.68 | 18 |
| 396 | CD16_RS04815 | 289.76 | 4 | 223.45 | 8 |
| 397 | CD16_RS03665 | 291.65 | 15 | 509.8 | 68 |
| 398 | CD16_RS03220 | 292.35 | 7 | 305.97 | 19 |
| 399 | CD16_RS03535 | 292.35 | 6 | 488.47 | 26 |
| 400 | gmk | 293.66 | 8 | 424.61 | 30 |
| 401 | sbcB | 294.82 | 17 | 213.98 | 32 |
| 402 | CD16_RS05550 | 294.98 | 4 | 625.57 | 22 |
| 403 | rsmG | 296.31 | 8 | 428.45 | 30 |
| 404 | dnaN | 296.89 | 14 | 392.49 | 48 |
| 405 | CD16_RS04670 | 297.66 | 12 | 363.45 | 38 |
| 406 | CD16_RS04455 | 297.66 | 4 | 631.25 | 22 |
| 407 | fliP | 298.26 | 9 | 204.45 | 16 |
| 408 | CD16_RS03090 | 298.75 | 10 | 449.25 | 39 |
| 409 | CD16_RS01840 | 299.48 | 3 | 307.93 | 8 |
| 410 | CD16_RS05415 | 300 | 7 | 380.07 | 23 |
| 411 | CD16_RS04000 | 300.94 | 10 | 348.12 | 30 |
| 412 | CD16_RS00485 | 301.58 | 7 | 232.57 | 14 |
| 413 | CD16_RS05585 | 303.17 | 4 | 350.7 | 12 |
| 414 | CD16_RS05250 | 303.84 | 17 | 365.25 | 53 |
| 415 | CD16_RS02205 | 304.3 | 10 | 469.33 | 40 |
| 416 | CD16_RS05260 | 304.58 | 8 | 381.69 | 26 |
| 417 | queC | 305.69 | 9 | 379.8 | 29 |
| 418 | flgK | 306.32 | 18 | 124.68 | 19 |
| 419 | nuoF | 307.44 | 16 | 407.5 | 55 |
| 420 | CD16_RS05130 | 308.25 | 9 | 158.47 | 12 |
| 421 | CD16_RS03320 | 310.06 | 5 | 406.49 | 17 |
| 422 | CD16_RS03070 | 310.49 | 11 | 282.98 | 26 |
| 423 | CD16_RS01345 | 311.12 | 29 | 264.75 | 64 |
| 424 | CD16_RS02585 | 312.17 | 9 | 334.35 | 25 |
| 425 | CD16_RS04990 | 314.31 | 23 | 389.92 | 74 |
| 426 | CD16_RS05590 | 315.64 | 15 | 438.15 | 54 |
| 427 | CD16_RS04965 | 318.1 | 15 | 629.62 | 77 |
| 428 | CD16_RS00145 | 318.51 | 10 | 356.16 | 29 |
| 429 | CD16_RS03115 | 321.01 | 4 | 556.99 | 18 |
| 430 | CD16_RS02655 | 321.01 | 6 | 763.28 | 37 |
| 431 | CD16_RS05090 | 321.53 | 24 | 299.61 | 58 |
| 432 | addB | 322.39 | 41 | 297.13 | 98 |
| 433 | CD16_RS00265 | 323.12 | 3 | 207.65 | 5 |
| 434 | CD16_RS00930 | 323.12 | 9 | 235.34 | 17 |
| 435 | CD16_RS03285 | 324.19 | 4 | 281.25 | 9 |
| 436 | CD16_RS02970 | 324.83 | 20 | 513.52 | 82 |
| 437 | CD16_RS02985 | 325.26 | 12 | 553.92 | 53 |
| 438 | CD16_RS03640 | 325.89 | 17 | 325.24 | 44 |
| 439 | CD16_RS04935 | 325.98 | 9 | 391.04 | 28 |
| 440 | CD16_RS04590 | 327.43 | 14 | 342.68 | 38 |
| 441 | CD16_RS03245 | 329.9 | 16 | 500.87 | 63 |
| 442 | CD16_RS00415 | 330.73 | 4 | 318.82 | 10 |
| 443 | CD16_RS00515 | 330.73 | 4 | 1,083.97 | 34 |
| 444 | CD16_RS03205 | 331.4 | 10 | 332.24 | 26 |
| 445 | CD16_RS05530 | 331.85 | 3 | 298.57 | 7 |
| 446 | CD16_RS04240 | 333.26 | 16 | 377.47 | 47 |
| 447 | CD16_RS00135 | 334.11 | 16 | 281.81 | 35 |
| 448 | CD16_RS02855 | 335.98 | 11 | 223.77 | 19 |
| 449 | uvrC | 336.22 | 26 | 548.48 | 110 |
| 450 | ribD | 336.4 | 15 | 276.71 | 32 |
| 451 | CD16_RS01420 | 339.89 | 12 | 436.85 | 40 |
| 452 | CD16_RS05195 | 341.07 | 19 | 214.57 | 31 |
| 453 | CD16_RS02325 | 341.07 | 9 | 336.08 | 23 |
| 454 | CD16_RS03120 | 341.07 | 8 | 526.05 | 32 |
| 455 | CD16_RS05465 | 341.07 | 17 | 572.46 | 74 |
| 456 | gatC | 341.07 | 4 | 986.34 | 30 |
| 457 | rfbB | 342.02 | 15 | 633.01 | 72 |
| 458 | CD16_RS04825 | 342.86 | 8 | 429.65 | 26 |
| 459 | CD16_RS03420 | 342.98 | 15 | 484.9 | 55 |
| 460 | CD16_RS02340 | 343.67 | 11 | 542.11 | 45 |
| 461 | bamA | 345.43 | 33 | 694.22 | 172 |
| 462 | CD16_RS00335 | 346.72 | 23 | 342.95 | 59 |
| 463 | CD16_RS03325 | 349.15 | 9 | 403.88 | 27 |
| 464 | CD16_RS03845 | 350.99 | 28 | 555.85 | 115 |
| 465 | CD16_RS04335 | 351.73 | 11 | 382.2 | 31 |
| 466 | CD16_RS01305 | 352.67 | 19 | 329.23 | 46 |
| 467 | CD16_RS03330 | 353.34 | 12 | 283.84 | 25 |
| 468 | mutS | 355.51 | 40 | 322.14 | 94 |
| 469 | ligA | 357.84 | 32 | 452.74 | 105 |
| 470 | CD16_RS01105 | 359.37 | 9 | 477.29 | 31 |
| 471 | rpmA | 359.81 | 4 | 936.48 | 27 |
| 472 | CD16_RS00400 | 361.8 | 8 | 627.77 | 36 |
| 473 | rfbA | 363.19 | 13 | 398.57 | 37 |
| 474 | CD16_RS04575 | 363.27 | 15 | 186.76 | 20 |
| 475 | CD16_RS01660 | 363.81 | 20 | 448.89 | 64 |
| 476 | CD16_RS01545 | 364.35 | 15 | 477.66 | 51 |
| 477 | pstC | 364.54 | 22 | 383.35 | 60 |
| 478 | CD16_RS04085 | 365.61 | 18 | 407.26 | 52 |
| 479 | uvrA | 366.65 | 43 | 272.89 | 83 |
| 480 | rplC | 368.72 | 10 | 668.22 | 47 |
| 481 | ubiE | 369.28 | 12 | 640.75 | 54 |
| 482 | glmU | 369.56 | 20 | 797.97 | 112 |
| 483 | CD16_RS00705 | 370.21 | 9 | 444.1 | 28 |
| 484 | CD16_RS04295 | 371.02 | 16 | 652.71 | 73 |
| 485 | CD16_RS04940 | 372.61 | 32 | 579.17 | 129 |
| 486 | recO | 373.62 | 11 | 366.7 | 28 |
| 487 | CD16_RS01165 | 374.51 | 14 | 484.79 | 47 |
| 488 | secB | 374.51 | 7 | 515.73 | 25 |
| 489 | CD16_RS00450 | 376.35 | 16 | 108.84 | 12 |
| 490 | lpxI | 377.35 | 13 | 425.31 | 38 |
| 491 | CD16_RS01970 | 377.44 | 16 | 409.31 | 45 |
| 492 | CD16_RS02990 | 377.44 | 16 | 454.79 | 50 |
| 493 | CD16_RS03910 | 377.8 | 3 | 0 | 0 |
| 494 | CD16_RS03215 | 378.97 | 5 | 1,110.54 | 38 |
| 495 | CD16_RS02245 | 379.93 | 22 | 346.26 | 52 |
| 496 | CD16_RS04230 | 381.84 | 32 | 464.7 | 101 |
| 497 | CD16_RS04640 | 381.89 | 23 | 486.57 | 76 |
| 498 | CD16_RS00775 | 382 | 14 | 147.29 | 14 |
| 499 | eno | 385.21 | 20 | 527.28 | 71 |
| 500 | CD16_RS03865 | 385.35 | 44 | 1,060.35 | 314 |
| 501 | CD16_RS05315 | 385.86 | 14 | 361.32 | 34 |
| 502 | uvrB | 385.93 | 38 | 258.45 | 66 |
| 503 | tilS | 386.12 | 20 | 468.97 | 63 |
| 504 | CD16_RS03230 | 386.34 | 16 | 661.05 | 71 |
| 505 | CD16_RS04585 | 387.49 | 16 | 196.1 | 21 |
| 506 | CD16_RS03335 | 388.37 | 13 | 506.85 | 44 |
| 507 | CD16_RS00975 | 388.37 | 13 | 702.67 | 61 |
| 508 | CD16_RS00215 | 389.79 | 19 | 822.69 | 104 |
| 509 | CD16_RS02845 | 390.72 | 20 | 203.39 | 27 |
| 510 | argC | 393.54 | 15 | 293.37 | 29 |
| 511 | rplD | 393.54 | 10 | 1,745.05 | 115 |
| 512 | CD16_RS00395 | 394.49 | 4 | 38.03 | 1 |
| 513 | CD16_RS02415 | 396.36 | 23 | 750.86 | 113 |
| 514 | CD16_RS00795 | 398.33 | 20 | 399.33 | 52 |
| 515 | flgF | 402.57 | 12 | 245.78 | 19 |
| 516 | CD16_RS01385 | 404.23 | 4 | 818.29 | 21 |
| 517 | CD16_RS01830 | 405.42 | 21 | 454.09 | 61 |
| 518 | CD16_RS01200 | 406.38 | 14 | 470.08 | 42 |
| 519 | CD16_RS05010 | 407.02 | 27 | 331.32 | 57 |
| 520 | CD16_RS05510 | 409.28 | 27 | 905.97 | 155 |
| 521 | CD16_RS01315 | 410.18 | 23 | 364.45 | 53 |
| 522 | CD16_RS03060 | 412.97 | 28 | 301.41 | 53 |
| 523 | carB | 415.27 | 59 | 797.89 | 294 |
| 524 | CD16_RS00050 | 415.61 | 23 | 613.14 | 88 |
| 525 | der | 417.1 | 24 | 629.91 | 94 |
| 526 | lpxA | 421.32 | 14 | 359.72 | 31 |
| 527 | CD16_RS04560 | 423.4 | 6 | 653.02 | 24 |
| 528 | CD16_RS05210 | 423.69 | 25 | 365.94 | 56 |
| 529 | CD16_RS04050 | 423.69 | 25 | 470.5 | 72 |
| 530 | CD16_RS01575 | 423.78 | 19 | 636.41 | 74 |
| 531 | accC | 424.03 | 23 | 561.59 | 79 |
| 532 | CD16_RS02160 | 424.44 | 14 | 233.8 | 20 |
| 533 | CD16_RS02515 | 424.62 | 18 | 463.89 | 51 |
| 534 | CD16_RS02035 | 426.02 | 42 | 551.47 | 141 |
| 535 | flaF | 427.08 | 6 | 164.68 | 6 |
| 536 | argF | 428.01 | 16 | 216.61 | 21 |
| 537 | hslV | 428.57 | 10 | 594.9 | 36 |
| 538 | CD16_RS02345 | 428.77 | 22 | 608.71 | 81 |
| 539 | CD16_RS04845 | 429.21 | 14 | 898.41 | 76 |
| 540 | CD16_RS05330 | 429.41 | 16 | 486.38 | 47 |
| 541 | CD16_RS04340 | 431.81 | 23 | 405.39 | 56 |
| 542 | flgD | 434.09 | 7 | 263.02 | 11 |
| 543 | coaD | 435.92 | 9 | 298.82 | 16 |
| 544 | uppS | 436.12 | 13 | 181.1 | 14 |
| 545 | CD16_RS03990 | 437.57 | 41 | 349.78 | 85 |
| 546 | topA | 439.57 | 45 | 485.87 | 129 |
| 547 | CD16_RS05080 | 440.09 | 10 | 593.92 | 35 |
| 548 | recN | 441.67 | 30 | 272.48 | 48 |
| 549 | purH | 442.06 | 29 | 775.84 | 132 |
| 550 | ulaA | 442.95 | 25 | 765.16 | 112 |
| 551 | CD16_RS02100 | 444.18 | 7 | 415.94 | 17 |
| 552 | gndA | 447.12 | 26 | 464.16 | 70 |
| 553 | CD16_RS01300 | 448.33 | 31 | 529.76 | 95 |
| 554 | nuoE | 448.53 | 12 | 662.96 | 46 |
| 555 | trmD | 449 | 13 | 426.16 | 32 |
| 556 | CD16_RS05450 | 449.76 | 5 | 485.58 | 14 |
| 557 | fliF | 449.92 | 31 | 324.58 | 58 |
| 558 | CD16_RS01655 | 450.59 | 12 | 593.61 | 41 |
| 559 | CD16_RS05575 | 454.76 | 5 | 245.49 | 7 |
| 560 | rsmH | 454.76 | 19 | 369.15 | 40 |
| 561 | CD16_RS03820 | 456.97 | 23 | 252.81 | 33 |
| 562 | CD16_RS03750 | 457.37 | 39 | 565.24 | 125 |
| 563 | rplK | 457.94 | 8 | 949.09 | 43 |
| 564 | CD16_RS02210 | 459.87 | 10 | 212.78 | 12 |
| 565 | CD16_RS03075 | 460.67 | 13 | 423.57 | 31 |
| 566 | CD16_RS02715 | 461.97 | 89 | 626.45 | 313 |
| 567 | CD16_RS04300 | 462.67 | 13 | 356.8 | 26 |
| 568 | CD16_RS00820 | 464.31 | 27 | 623.3 | 94 |
| 569 | CD16_RS05030 | 465.41 | 17 | 285.01 | 27 |
| 570 | CD16_RS00920 | 465.85 | 7 | 538.88 | 21 |
| 571 | dnaQ | 465.85 | 14 | 564.54 | 44 |
| 572 | rplN | 465.85 | 7 | 667.18 | 26 |
| 573 | CD16_RS05470 | 466.82 | 43 | 816.28 | 195 |
| 574 | CD16_RS00455 | 470.86 | 13 | 432.94 | 31 |
| 575 | CD16_RS02880 | 472.95 | 13 | 196.39 | 14 |
| 576 | CD16_RS01230 | 473.16 | 10 | 273.67 | 15 |
| 577 | CD16_RS02400 | 473.55 | 7 | 156.51 | 6 |
| 578 | CD16_RS01835 | 474.17 | 19 | 500.38 | 52 |
| 579 | CD16_RS02270 | 478.38 | 18 | 358.67 | 35 |
| 580 | gorA | 478.38 | 27 | 403.07 | 59 |
| 581 | CD16_RS03520 | 478.38 | 27 | 840.31 | 123 |
| 582 | CD16_RS01435 | 478.58 | 48 | 768.88 | 200 |
| 583 | CD16_RS01705 | 478.95 | 22 | 386.14 | 46 |
| 584 | CD16_RS02040 | 479.16 | 12 | 369.51 | 24 |
| 585 | mnmA | 480.33 | 24 | 717.69 | 93 |
| 586 | CD16_RS03880 | 481.51 | 6 | 309.44 | 10 |
| 587 | queD | 481.51 | 7 | 397.85 | 15 |
| 588 | purD | 481.51 | 25 | 616.4 | 83 |
| 589 | rnhA | 481.51 | 9 | 763.28 | 37 |
| 590 | purQ | 483.7 | 13 | 702.99 | 49 |
| 591 | CD16_RS01720 | 484.01 | 57 | 455.11 | 139 |
| 592 | CD16_RS03110 | 484.51 | 19 | 530.96 | 54 |
| 593 | CD16_RS00570 | 484.68 | 9 | 124.59 | 6 |
| 594 | miaA | 484.68 | 18 | 446.45 | 43 |
| 595 | CD16_RS03975 | 484.8 | 26 | 603.93 | 84 |
| 596 | dcm | 487.24 | 5 | 187.87 | 5 |
| 597 | CD16_RS02755 | 489.92 | 24 | 590.33 | 75 |
| 598 | CD16_RS04525 | 491.14 | 6 | 441.88 | 14 |
| 599 | CD16_RS02965 | 491.52 | 26 | 553.99 | 76 |
| 600 | nuoH | 492.55 | 21 | 370.79 | 41 |
| 601 | CD16_RS00765 | 492.55 | 21 | 624.02 | 69 |
| 602 | CD16_RS01820 | 492.86 | 23 | 578.37 | 70 |
| 603 | CD16_RS00205 | 494.15 | 23 | 695.87 | 84 |
| 604 | serB | 494.44 | 18 | 444.84 | 42 |
| 605 | mutL | 495.27 | 36 | 493.33 | 93 |
| 606 | CD16_RS01335 | 497.47 | 22 | 619.05 | 71 |
| 607 | zwf | 498.11 | 30 | 352.12 | 55 |
| 608 | CD16_RS01860 | 499.2 | 57 | 1,087.36 | 322 |
| 609 | CD16_RS03590 | 499.2 | 57 | 1,090.74 | 323 |
| 610 | CD16_RS02460 | 499.47 | 18 | 374.47 | 35 |
| 611 | mnmG | 499.55 | 38 | 643.76 | 127 |
| 612 | CD16_RS01410 | 500.09 | 19 | 740.86 | 73 |
| 613 | CD16_RS04605 | 501.47 | 34 | 608.51 | 107 |
| 614 | CD16_RS03605 | 502.63 | 21 | 535.27 | 58 |
| 615 | CD16_RS03195 | 503.03 | 22 | 740.58 | 84 |
| 616 | CD16_RS02645 | 503.73 | 8 | 339.91 | 14 |
| 617 | mraZ | 504.6 | 9 | 302.66 | 14 |
| 618 | murD | 506.15 | 29 | 336.49 | 50 |
| 619 | CD16_RS01295 | 508.08 | 27 | 413.58 | 57 |
| 620 | CD16_RS03300 | 508.43 | 10 | 725.35 | 37 |
| 621 | CD16_RS02405 | 508.71 | 11 | 588.46 | 33 |
| 622 | purU | 509.83 | 18 | 535.15 | 49 |
| 623 | CD16_RS04615 | 511.61 | 4 | 0 | 0 |
| 624 | parE | 512.35 | 43 | 776.43 | 169 |
| 625 | argH | 512.67 | 30 | 560.09 | 85 |
| 626 | glnA | 517.18 | 29 | 508.85 | 74 |
| 627 | bcp | 518.08 | 10 | 779.08 | 39 |
| 628 | mnmE | 519.73 | 28 | 543.94 | 76 |
| 629 | CD16_RS05490 | 520.48 | 11 | 437.86 | 24 |
| 630 | CD16_RS01075 | 522.49 | 18 | 380.54 | 34 |
| 631 | pgl | 522.49 | 15 | 564.1 | 42 |
| 632 | hemB | 523.5 | 22 | 660.62 | 72 |
| 633 | CD16_RS01125 | 523.5 | 11 | 1,027.62 | 56 |
| 634 | CD16_RS04180 | 524.72 | 20 | 546.28 | 54 |
| 635 | CD16_RS00040 | 525.28 | 24 | 438.84 | 52 |
| 636 | CD16_RS00560 | 526.8 | 13 | 765.63 | 49 |
| 637 | nuoL | 527.71 | 43 | 695.61 | 147 |
| 638 | CD16_RS00985 | 528.11 | 4 | 407.26 | 8 |
| 639 | CD16_RS00945 | 528.11 | 52 | 567.82 | 145 |
| 640 | CD16_RS01445 | 531.13 | 34 | 819.19 | 136 |
| 641 | CD16_RS02290 | 533.37 | 24 | 419.88 | 49 |
| 642 | carA | 536.09 | 26 | 874.53 | 110 |
| 643 | miaB | 536.18 | 30 | 723.6 | 105 |
| 644 | CD16_RS03185 | 536.77 | 12 | 534.67 | 31 |
| 645 | CD16_RS04495 | 536.77 | 20 | 538.12 | 52 |
| 646 | CD16_RS02420 | 536.77 | 16 | 1,060.71 | 82 |
| 647 | nth | 538.53 | 15 | 595.26 | 43 |
| 648 | ybeY | 539.18 | 11 | 378 | 20 |
| 649 | CD16_RS05175 | 540.17 | 13 | 208.28 | 13 |
| 650 | CD16_RS02675 | 542.21 | 31 | 762.09 | 113 |
| 651 | parC | 542.82 | 50 | 611.16 | 146 |
| 652 | pyk | 544.58 | 32 | 866.17 | 132 |
| 653 | rpsJ | 545.71 | 7 | 691.37 | 23 |
| 654 | CD16_RS05460 | 545.71 | 54 | 935.19 | 240 |
| 655 | CD16_RS01605 | 550.96 | 21 | 424.88 | 42 |
| 656 | CD16_RS02835 | 550.96 | 14 | 485.58 | 32 |
| 657 | CD16_RS01025 | 551.12 | 34 | 306.25 | 49 |
| 658 | CD16_RS03080 | 551.84 | 18 | 413.74 | 35 |
| 659 | CD16_RS03140 | 551.84 | 18 | 567.42 | 48 |
| 660 | tsaE | 552.41 | 11 | 484.09 | 25 |
| 661 | lysS | 553.31 | 34 | 414.14 | 66 |
| 662 | CD16_RS02760 | 553.74 | 23 | 919.03 | 99 |
| 663 | purL | 554.59 | 50 | 735.61 | 172 |
| 664 | CD16_RS01775 | 555.82 | 11 | 779.33 | 40 |
| 665 | CD16_RS04530 | 555.89 | 51 | 374.05 | 89 |
| 666 | CD16_RS01180 | 556.01 | 18 | 404.96 | 34 |
| 667 | plsY | 556.31 | 14 | 490.29 | 32 |
| 668 | CD16_RS03735 | 556.71 | 27 | 675.78 | 85 |
| 669 | sufA | 559.7 | 8 | 269.77 | 10 |
| 670 | CD16_RS03000 | 560.36 | 51 | 652.44 | 154 |
| 671 | CD16_RS05335 | 561.76 | 21 | 835.48 | 81 |
| 672 | CD16_RS04775 | 563.14 | 28 | 511.83 | 66 |
| 673 | rirA | 564.53 | 10 | 522.42 | 24 |
| 674 | rplA | 564.53 | 16 | 585 | 43 |
| 675 | CD16_RS02735 | 565.56 | 19 | 183.64 | 16 |
| 676 | queA | 566.88 | 25 | 620.76 | 71 |
| 677 | CD16_RS01925 | 571.09 | 30 | 719.34 | 98 |
| 678 | recA | 571.63 | 25 | 652.41 | 74 |
| 679 | prfA | 571.63 | 25 | 855.19 | 97 |
| 680 | trmA | 572.12 | 26 | 237.57 | 28 |
| 681 | dapD | 572.43 | 20 | 860.8 | 78 |
| 682 | rph | 572.66 | 17 | 428.63 | 33 |
| 683 | CD16_RS02765 | 574.43 | 12 | 959.8 | 52 |
| 684 | rfbC | 575.88 | 14 | 761.31 | 48 |
| 685 | CD16_RS04055 | 575.88 | 42 | 819.47 | 155 |
| 686 | ubiG | 577.81 | 18 | 742.65 | 60 |
| 687 | polA | 578.11 | 69 | 613.81 | 190 |
| 688 | CD16_RS03015 | 578.49 | 20 | 289.98 | 26 |
| 689 | CD16_RS03680 | 579.72 | 25 | 527.54 | 59 |
| 690 | CD16_RS03635 | 582.09 | 48 | 607.87 | 130 |
| 691 | CD16_RS04995 | 584.69 | 9 | 501 | 20 |
| 692 | rpmG | 584.69 | 4 | 619.98 | 11 |
| 693 | CD16_RS01525 | 594.49 | 13 | 652.41 | 37 |
| 694 | rlmN | 595.32 | 28 | 385.31 | 47 |
| 695 | lipA | 595.32 | 24 | 468.66 | 49 |
| 696 | CD16_RS02310 | 595.32 | 12 | 1,013.83 | 53 |
| 697 | sucD | 598.29 | 22 | 912.28 | 87 |
| 698 | CD16_RS04235 | 600 | 14 | 214.82 | 13 |
| 699 | CD16_RS01100 | 600.28 | 11 | 652.3 | 31 |
| 700 | CD16_RS02115 | 600.28 | 11 | 673.34 | 32 |
| 701 | thrS | 601.7 | 48 | 420.51 | 87 |
| 702 | rsmD | 603.16 | 14 | 398.69 | 24 |
| 703 | CD16_RS03720 | 603.86 | 9 | 983.1 | 38 |
| 704 | CD16_RS03630 | 606.35 | 24 | 769.58 | 79 |
| 705 | CD16_RS02230 | 606.35 | 8 | 1,373.56 | 47 |
| 706 | CD16_RS04175 | 610.34 | 17 | 539.89 | 39 |
| 707 | CD16_RS04285 | 610.87 | 15 | 518.19 | 33 |
| 708 | CD16_RS00505 | 610.94 | 133 | 524.27 | 296 |
| 709 | CD16_RS00760 | 611.74 | 21 | 561.61 | 50 |
| 710 | CD16_RS00220 | 613.4 | 29 | 921.6 | 113 |
| 711 | CD16_RS04315 | 613.93 | 6 | 591.8 | 15 |
| 712 | rpsB | 616.13 | 21 | 1,300.97 | 115 |
| 713 | hflK | 619.09 | 27 | 866.43 | 98 |
| 714 | CD16_RS00865 | 619.74 | 24 | 846.32 | 85 |
| 715 | cyoC | 620.72 | 16 | 568.43 | 38 |
| 716 | CD16_RS01490 | 620.98 | 11 | 740.09 | 34 |
| 717 | CD16_RS04270 | 621.36 | 23 | 687.5 | 66 |
| 718 | CD16_RS00200 | 621.7 | 6 | 239.72 | 6 |
| 719 | CD16_RS02220 | 621.7 | 30 | 655.23 | 82 |
| 720 | CD16_RS03780 | 622.11 | 38 | 549.19 | 87 |
| 721 | sufC | 622.11 | 19 | 871.13 | 69 |
| 722 | CD16_RS05595 | 622.49 | 20 | 468.04 | 39 |
| 723 | CD16_RS05085 | 624.02 | 17 | 325.53 | 23 |
| 724 | sdhD | 624.86 | 10 | 650.53 | 27 |
| 725 | CD16_RS01040 | 625.66 | 12 | 201.04 | 10 |
| 726 | rpsG | 625.66 | 12 | 321.66 | 16 |
| 727 | CD16_RS03145 | 629.67 | 16 | 789.07 | 52 |
| 728 | CD16_RS03570 | 631.61 | 25 | 779.33 | 80 |
| 729 | CD16_RS00495 | 632.81 | 31 | 936.65 | 119 |
| 730 | secA | 633.91 | 69 | 807.67 | 228 |
| 731 | CD16_RS01325 | 634.1 | 11 | 489 | 22 |
| 732 | CD16_RS03800 | 636.66 | 35 | 680.35 | 97 |
| 733 | murJ_2 | 638.45 | 41 | 558.4 | 93 |
| 734 | CD16_RS04985 | 641.05 | 39 | 443.65 | 70 |
| 735 | CD16_RS01210 | 641.51 | 25 | 435.35 | 44 |
| 736 | CD16_RS04540 | 643.42 | 18 | 882.1 | 64 |
| 737 | nhaA | 644.05 | 31 | 272.37 | 34 |
| 738 | CD16_RS00870 | 644.24 | 34 | 621.03 | 85 |
| 739 | glmS | 645.18 | 48 | 673.75 | 130 |
| 740 | rplB | 645.47 | 22 | 1,153.91 | 102 |
| 741 | infB | 647.45 | 70 | 616.99 | 173 |
| 742 | CD16_RS04255 | 648.68 | 21 | 512.15 | 43 |
| 743 | dapE | 650.66 | 31 | 963.07 | 119 |
| 744 | sufB | 651.51 | 39 | 534.63 | 83 |
| 745 | gyrB | 651.6 | 64 | 777.29 | 198 |
| 746 | dusA | 652.99 | 28 | 494.57 | 55 |
| 747 | CD16_RS03030 | 653.22 | 32 | 1,031.10 | 131 |
| 748 | CD16_RS03985 | 654.06 | 33 | 1,123.42 | 147 |
| 749 | CD16_RS00300 | 655.9 | 25 | 485.58 | 48 |
| 750 | CD16_RS03795 | 657.78 | 45 | 681.98 | 121 |
| 751 | guaA | 659.88 | 42 | 836.02 | 138 |
| 752 | CD16_RS01080 | 660.14 | 25 | 1,048.70 | 103 |
| 753 | alaS | 664.69 | 73 | 800.48 | 228 |
| 754 | argJ | 667.42 | 34 | 401.16 | 53 |
| 755 | rpmB | 668.22 | 8 | 515.31 | 16 |
| 756 | prmC | 668.22 | 24 | 676.34 | 63 |
| 757 | CD16_RS03380 | 668.59 | 37 | 195.09 | 28 |
| 758 | gshB | 671.38 | 26 | 627.27 | 63 |
| 759 | hemA | 671.96 | 33 | 620.26 | 79 |
| 760 | CD16_RS02330 | 674.64 | 15 | 1,335.35 | 77 |
| 761 | CD16_RS00510 | 675.11 | 8 | 650.78 | 20 |
| 762 | CD16_RS00475 | 680.25 | 30 | 944.26 | 108 |
| 763 | rsfS | 682.14 | 10 | 604.95 | 23 |
| 764 | murA | 683.72 | 36 | 519.94 | 71 |
| 765 | CD16_RS04220 | 683.77 | 35 | 580.03 | 77 |
| 766 | CD16_RS02070 | 683.77 | 35 | 602.63 | 80 |
| 767 | CD16_RS00480 | 683.92 | 32 | 964.19 | 117 |
| 768 | pyrF | 684.99 | 20 | 660.31 | 50 |
| 769 | typA | 685.5 | 51 | 751.49 | 145 |
| 770 | sufD | 686.91 | 36 | 595.94 | 81 |
| 771 | CD16_RS02425 | 688.52 | 36 | 464.59 | 63 |
| 772 | CD16_RS01700 | 696.65 | 40 | 537.24 | 80 |
| 773 | CD16_RS03160 | 698.11 | 51 | 575.31 | 109 |
| 774 | ftsZ | 699.77 | 43 | 734.16 | 117 |
| 775 | CD16_RS02355 | 700.58 | 19 | 497.61 | 35 |
| 776 | lysA | 701.09 | 37 | 818.29 | 112 |
| 777 | CD16_RS04850 | 701.63 | 18 | 255.51 | 17 |
| 778 | CD16_RS05440 | 701.63 | 27 | 791.57 | 79 |
| 779 | CD16_RS05455 | 704.59 | 34 | 878.96 | 110 |
| 780 | CD16_RS02075 | 710.46 | 46 | 476.42 | 80 |
| 781 | pstA | 710.96 | 37 | 489 | 66 |
| 782 | CD16_RS00370 | 711.8 | 14 | 744.96 | 38 |
| 783 | CD16_RS00375 | 713.87 | 30 | 495.46 | 54 |
| 784 | lptB | 715.86 | 23 | 540.05 | 45 |
| 785 | CD16_RS00195 | 718.59 | 23 | 819.19 | 68 |
| 786 | lpdA_2 | 718.66 | 41 | 655.59 | 97 |
| 787 | CD16_RS02505 | 718.74 | 18 | 816.01 | 53 |
| 788 | CD16_RS02365 | 721.02 | 17 | 981.22 | 60 |
| 789 | rpmC | 722.27 | 6 | 324.91 | 7 |
| 790 | CD16_RS04705 | 727.62 | 36 | 623.46 | 80 |
| 791 | CD16_RS03610 | 728.77 | 56 | 546.95 | 109 |
| 792 | gatA | 729.09 | 44 | 920.05 | 144 |
| 793 | nusA | 730.03 | 47 | 1,018.15 | 170 |
| 794 | CD16_RS01685 | 733.05 | 24 | 753.74 | 64 |
| 795 | gatB | 735.24 | 45 | 686.69 | 109 |
| 796 | pheS | 736.04 | 33 | 662.22 | 77 |
| 797 | CD16_RS03765 | 736.71 | 18 | 883.76 | 56 |
| 798 | CD16_RS01965 | 736.71 | 18 | 1,357.20 | 86 |
| 799 | CD16_RS01955 | 737.77 | 21 | 826.32 | 61 |
| 800 | CD16_RS05635 | 738.13 | 52 | 301.03 | 55 |
| 801 | CD16_RS04905 | 739.95 | 16 | 838.11 | 47 |
| 802 | CD16_RS03730 | 740.41 | 18 | 777.17 | 49 |
| 803 | CD16_RS00365 | 741.34 | 24 | 881.37 | 74 |
| 804 | CD16_RS01390 | 741.56 | 26 | 692.84 | 63 |
| 805 | ftsA | 742.47 | 40 | 601.19 | 84 |
| 806 | cpaB | 744.15 | 24 | 430.4 | 36 |
| 807 | bioB | 746.41 | 30 | 1,112.85 | 116 |
| 808 | CD16_RS02455 | 747.24 | 22 | 261.93 | 20 |
| 809 | CD16_RS03100 | 750.35 | 22 | 604.95 | 46 |
| 810 | CD16_RS04700 | 753.28 | 15 | 387.27 | 20 |
| 811 | CD16_RS02080 | 755.6 | 12 | 388.46 | 16 |
| 812 | mltG | 762.03 | 31 | 473.91 | 50 |
| 813 | grxD | 765.02 | 10 | 1,386.40 | 47 |
| 814 | CD16_RS05060 | 765.33 | 46 | 872.47 | 136 |
| 815 | CD16_RS03660 | 765.41 | 36 | 557.47 | 68 |
| 816 | CD16_RS03315 | 765.7 | 42 | 850.58 | 121 |
| 817 | nusB | 765.91 | 16 | 812.14 | 44 |
| 818 | coaBC | 766.15 | 38 | 909.57 | 117 |
| 819 | tuf_1 | 770.66 | 37 | 1,276.97 | 159 |
| 820 | rimM | 771.43 | 18 | 991.5 | 60 |
| 821 | CD16_RS03135 | 774.78 | 23 | 753.35 | 58 |
| 822 | CD16_RS02945 | 776.95 | 28 | 716.85 | 67 |
| 823 | rpsE | 777.64 | 19 | 820.63 | 52 |
| 824 | CD16_RS02435 | 778.39 | 31 | 890.73 | 92 |
| 825 | ffh | 779.59 | 44 | 1,099.91 | 161 |
| 826 | CD16_RS02295 | 780.54 | 78 | 760.13 | 197 |
| 827 | CD16_RS03290 | 782.45 | 26 | 858.69 | 74 |
| 828 | CD16_RS03550 | 782.98 | 11 | 521.47 | 19 |
| 829 | CD16_RS04045 | 783.32 | 60 | 1,328.96 | 264 |
| 830 | CD16_RS04410 | 784.93 | 7 | 259.42 | 6 |
| 831 | CD16_RS05495 | 785.25 | 33 | 1,321.23 | 144 |
| 832 | CD16_RS03930 | 794.13 | 13 | 1,201.27 | 51 |
| 833 | murG | 796.44 | 36 | 605.66 | 71 |
| 834 | CD16_RS04150 | 797.88 | 27 | 991.32 | 87 |
| 835 | nuoI | 798.6 | 16 | 808.31 | 42 |
| 836 | folD | 799.9 | 30 | 781.36 | 76 |
| 837 | CD16_RS04910 | 800.77 | 9 | 480.3 | 14 |
| 838 | CD16_RS02530 | 802.52 | 25 | 1,076.85 | 87 |
| 839 | ettA | 803.41 | 53 | 835.83 | 143 |
| 840 | CD16_RS03180 | 805.84 | 19 | 1,308.30 | 80 |
| 841 | CD16_RS02025 | 807.56 | 22 | 622.76 | 44 |
| 842 | CD16_RS04860 | 808.46 | 24 | 1,026.11 | 79 |
| 843 | CD16_RS03965 | 809.19 | 23 | 705.41 | 52 |
| 844 | clpA | 809.35 | 79 | 612.29 | 155 |
| 845 | CD16_RS01055 | 809.57 | 18 | 502.92 | 29 |
| 846 | CD16_RS01485 | 810.66 | 41 | 1,303.68 | 171 |
| 847 | CD16_RS03955 | 811.66 | 47 | 1,005.48 | 151 |
| 848 | guaB | 811.94 | 49 | 888.1 | 139 |
| 849 | CD16_RS03170 | 814.5 | 20 | 863.66 | 55 |
| 850 | secD | 814.64 | 83 | 825.02 | 218 |
| 851 | CD16_RS02450 | 815.43 | 26 | 604.65 | 50 |
| 852 | CD16_RS00860 | 815.6 | 55 | 526.05 | 92 |
| 853 | CD16_RS01070 | 820.91 | 35 | 488.36 | 54 |
| 854 | CD16_RS01550 | 821.31 | 30 | 1,002.83 | 95 |
| 855 | CD16_RS02350 | 823.02 | 37 | 720.45 | 84 |
| 856 | lipB | 824.91 | 26 | 1,076.56 | 88 |
| 857 | ruvA | 826.44 | 21 | 834.59 | 55 |
| 858 | CD16_RS01930 | 832.93 | 29 | 1,129.61 | 102 |
| 859 | rpoH | 837.48 | 31 | 1,364.59 | 131 |
| 860 | CD16_RS00745 | 838.12 | 30 | 991.05 | 92 |
| 861 | secG | 844.55 | 13 | 576.14 | 23 |
| 862 | CD16_RS03340 | 844.79 | 29 | 662.7 | 59 |
| 863 | CD16_RS01785 | 845.26 | 38 | 737.61 | 86 |
| 864 | tuf_2 | 853.98 | 41 | 1,076.18 | 134 |
| 865 | CD16_RS05525 | 854.55 | 19 | 971.16 | 56 |
| 866 | CD16_RS00695 | 856.64 | 18 | 1,724.94 | 94 |
| 867 | cyoA | 860.36 | 35 | 1,326.96 | 140 |
| 868 | CD16_RS04420 | 861.65 | 16 | 477.59 | 23 |
| 869 | CD16_RS02300 | 870.58 | 77 | 967.81 | 222 |
| 870 | CD16_RS04535 | 877.04 | 21 | 837.38 | 52 |
| 871 | CD16_RS03165 | 877.96 | 34 | 746.75 | 75 |
| 872 | CD16_RS01770 | 878.29 | 25 | 623.13 | 46 |
| 873 | CD16_RS04915 | 880.53 | 27 | 641.31 | 51 |
| 874 | CD16_RS02670 | 888.07 | 46 | 982.61 | 132 |
| 875 | CD16_RS01120 | 890.57 | 47 | 905.97 | 124 |
| 876 | CD16_RS02285 | 895.79 | 29 | 762.27 | 64 |
| 877 | CD16_RS02590 | 897.06 | 8 | 475.6 | 11 |
| 878 | CD16_RS02940 | 898.82 | 56 | 742.65 | 120 |
| 879 | CD16_RS03685 | 906.72 | 36 | 631.25 | 65 |
| 880 | CD16_RS02380 | 909.52 | 7 | 150.3 | 3 |
| 881 | CD16_RS02865 | 909.52 | 7 | 350.7 | 7 |
| 882 | CD16_RS04695 | 913.02 | 29 | 704.09 | 58 |
| 883 | CD16_RS01895 | 914.87 | 57 | 643.63 | 104 |
| 884 | CD16_RS01205 | 916.8 | 28 | 694.38 | 55 |
| 885 | CD16_RS02555 | 918.8 | 11 | 289.86 | 9 |
| 886 | ppa | 919.74 | 20 | 762.47 | 43 |
| 887 | rpsQ | 920.89 | 9 | 1,144.15 | 29 |
| 888 | rplE | 924.19 | 21 | 610.89 | 36 |
| 889 | CD16_RS05445 | 926.68 | 18 | 218.36 | 11 |
| 890 | CD16_RS00165 | 928.27 | 11 | 488.08 | 15 |
| 891 | rpsM | 931.7 | 14 | 846.8 | 33 |
| 892 | CD16_RS04625 | 935.38 | 209 | 792.09 | 459 |
| 893 | tsf | 937.08 | 34 | 1,179.62 | 111 |
| 894 | acnA | 939.94 | 103 | 876.16 | 249 |
| 895 | CD16_RS03155 | 948.56 | 27 | 1,815.20 | 134 |
| 896 | rplP | 949.06 | 16 | 1,440.91 | 63 |
| 897 | CD16_RS01140 | 951.82 | 50 | 1,012.94 | 138 |
| 898 | CD16_RS02550 | 952.21 | 57 | 1,056.39 | 164 |
| 899 | fumC | 952.64 | 54 | 741.45 | 109 |
| 900 | CD16_RS01695 | 963.02 | 50 | 913.46 | 123 |
| 901 | nusG | 965.73 | 21 | 1,241.23 | 70 |
| 902 | CD16_RS01980 | 972.17 | 50 | 742.21 | 99 |
| 903 | CD16_RS04445 | 972.25 | 31 | 1,330.23 | 110 |
| 904 | CD16_RS00730 | 978.16 | 38 | 823.81 | 83 |
| 905 | CD16_RS03690 | 983.44 | 34 | 881.08 | 79 |
| 906 | CD16_RS00225 | 983.55 | 31 | 1,260.06 | 103 |
| 907 | lpxD | 987.93 | 42 | 780 | 86 |
| 908 | CD16_RS04955 | 999.51 | 58 | 784.08 | 118 |
| 909 | clpS | 1,001.13 | 17 | 999.11 | 44 |
| 910 | CD16_RS03310 | 1,004.61 | 81 | 870.37 | 182 |
| 911 | rplF | 1,011.71 | 22 | 1,773.19 | 100 |
| 912 | CD16_RS01530 | 1,020.61 | 49 | 610.37 | 76 |
| 913 | CD16_RS03950 | 1,023.21 | 44 | 833.9 | 93 |
| 914 | CD16_RS00245 | 1,023.21 | 15 | 867.97 | 33 |
| 915 | atpC | 1,023.21 | 17 | 1,253.23 | 54 |
| 916 | CD16_RS00440 | 1,027.10 | 33 | 864.07 | 72 |
| 917 | CD16_RS00235 | 1,033.14 | 13 | 1,378.95 | 45 |
| 918 | CD16_RS05355 | 1,033.98 | 12 | 863.82 | 26 |
| 919 | CD16_RS03790 | 1,035.34 | 32 | 648.72 | 52 |
| 920 | tkt | 1,038.67 | 84 | 1,039.38 | 218 |
| 921 | rpsN | 1,043.27 | 13 | 773.6 | 25 |
| 922 | serS | 1,044.58 | 55 | 988.62 | 135 |
| 923 | CD16_RS04305 | 1,046.29 | 51 | 648.66 | 82 |
| 924 | CD16_RS04390 | 1,048.41 | 26 | 870.7 | 56 |
| 925 | pdhA | 1,049.45 | 45 | 971.16 | 108 |
| 926 | CD16_RS00530 | 1,051.00 | 52 | 1,246.92 | 160 |
| 927 | lepB | 1,051.98 | 32 | 849.28 | 67 |
| 928 | rseP | 1,052.44 | 45 | 568.13 | 63 |
| 929 | CD16_RS00405 | 1,052.82 | 40 | 537.89 | 53 |
| 930 | CD16_RS03725 | 1,056.22 | 24 | 899.37 | 53 |
| 931 | CD16_RS05255 | 1,058.01 | 19 | 794.44 | 37 |
| 932 | CD16_RS04690 | 1,070.02 | 80 | 1,351.21 | 262 |
| 933 | CD16_RS01275 | 1,071.52 | 61 | 1,496.86 | 221 |
| 934 | ssb | 1,074.37 | 21 | 867.97 | 44 |
| 935 | CD16_RS01690 | 1,080.90 | 89 | 1,311.21 | 280 |
| 936 | CD16_RS00355 | 1,081.41 | 72 | 1,106.14 | 191 |
| 937 | yidC | 1,082.99 | 77 | 1,073.78 | 198 |
| 938 | CD16_RS03085 | 1,083.40 | 36 | 1,032.75 | 89 |
| 939 | folE | 1,085.86 | 26 | 676.34 | 42 |
| 940 | ribB | 1,086.25 | 28 | 1,091.98 | 73 |
| 941 | frr | 1,094.34 | 25 | 1,147.74 | 68 |
| 942 | CD16_RS02130 | 1,101.92 | 21 | 1,092.56 | 54 |
| 943 | gap | 1,102.86 | 45 | 973.34 | 103 |
| 944 | CD16_RS02020 | 1,108.12 | 118 | 869.03 | 240 |
| 945 | dctA | 1,111.87 | 58 | 1,086.59 | 147 |
| 946 | CD16_RS00690 | 1,113.25 | 17 | 2,348.27 | 93 |
| 947 | CD16_RS00435 | 1,113.49 | 37 | 974.73 | 84 |
| 948 | CD16_RS00890 | 1,120.66 | 46 | 817.25 | 87 |
| 949 | hslU | 1,121.33 | 60 | 951.21 | 132 |
| 950 | rnpA | 1,122.23 | 17 | 890.88 | 35 |
| 951 | CD16_RS01665 | 1,133.40 | 18 | 1,044.00 | 43 |
| 952 | CD16_RS04725 | 1,136.43 | 133 | 1,367.28 | 415 |
| 953 | CD16_RS01610 | 1,138.88 | 64 | 1,372.29 | 200 |
| 954 | CD16_RS02440 | 1,141.03 | 46 | 927.75 | 97 |
| 955 | sucC | 1,148.87 | 56 | 1,218.21 | 154 |
| 956 | CD16_RS05515 | 1,155.81 | 73 | 1,153.84 | 189 |
| 957 | CD16_RS01590 | 1,157.33 | 32 | 627.54 | 45 |
| 958 | cyoD | 1,159.64 | 17 | 894.28 | 34 |
| 959 | CD16_RS05480 | 1,167.35 | 82 | 1,558.92 | 284 |
| 960 | phoU | 1,174.47 | 33 | 1,454.63 | 106 |
| 961 | secY | 1,177.27 | 64 | 1,007.17 | 142 |
| 962 | CD16_RS05430 | 1,191.59 | 23 | 1,118.68 | 56 |
| 963 | CD16_RS03345 | 1,193.17 | 43 | 481.47 | 45 |
| 964 | CD16_RS00785 | 1,193.75 | 91 | 925.64 | 183 |
| 965 | CD16_RS04260 | 1,202.42 | 26 | 499.3 | 28 |
| 966 | CD16_RS04975 | 1,204.43 | 54 | 662.22 | 77 |
| 967 | CD16_RS01425 | 1,206.31 | 14 | 99.67 | 3 |
| 968 | CD16_RS01520 | 1,206.31 | 28 | 830.6 | 50 |
| 969 | atpD | 1,213.33 | 71 | 1,436.47 | 218 |
| 970 | lon | 1,216.39 | 122 | 1,237.90 | 322 |
| 971 | odhB | 1,217.55 | 65 | 895.6 | 124 |
| 972 | CD16_RS03960 | 1,219.98 | 31 | 940.81 | 62 |
| 973 | lpdA_1 | 1,239.74 | 73 | 877.47 | 134 |
| 974 | CD16_RS00770 | 1,256.22 | 31 | 921.88 | 59 |
| 975 | rpoC | 1,257.99 | 215 | 1,477.74 | 655 |
| 976 | CD16_RS05500 | 1,259.34 | 38 | 1,686.75 | 132 |
| 977 | CD16_RS03525 | 1,259.99 | 147 | 1,718.60 | 520 |
| 978 | CD16_RS04140 | 1,260.66 | 146 | 1,102.03 | 331 |
| 979 | CD16_RS05295 | 1,263.82 | 65 | 1,454.43 | 194 |
| 980 | CD16_RS02430 | 1,268.44 | 75 | 710.81 | 109 |
| 981 | CD16_RS00035 | 1,269.83 | 121 | 1,128.97 | 279 |
| 982 | surE | 1,271.88 | 39 | 1,408.38 | 112 |
| 983 | rplI | 1,273.33 | 28 | 2,419.81 | 138 |
| 984 | CD16_RS04425 | 1,276.26 | 29 | 933.31 | 55 |
| 985 | CD16_RS04245 | 1,277.76 | 32 | 615.86 | 40 |
| 986 | rpoD | 1,282.38 | 107 | 1,381.74 | 299 |
| 987 | CD16_RS01595 | 1,290.69 | 152 | 939.68 | 287 |
| 988 | CD16_RS01500 | 1,300.53 | 51 | 766.94 | 78 |
| 989 | CD16_RS03545 | 1,309.71 | 52 | 844.91 | 87 |
| 990 | CD16_RS04060 | 1,311.81 | 100 | 1,411.22 | 279 |
| 991 | CD16_RS02545 | 1,320.27 | 80 | 1,151.78 | 181 |
| 992 | CD16_RS00545 | 1,320.27 | 55 | 1,323.60 | 143 |
| 993 | CD16_RS01170 | 1,320.27 | 10 | 1,476.32 | 29 |
| 994 | sdhC | 1,322.30 | 21 | 971.16 | 40 |
| 995 | CD16_RS01330 | 1,322.94 | 80 | 1,893.76 | 297 |
| 996 | plsX | 1,323.16 | 59 | 838.79 | 97 |
| 997 | mscL | 1,325.85 | 23 | 866.86 | 39 |
| 998 | clpB | 1,341.91 | 140 | 1,223.33 | 331 |
| 999 | lepA | 1,348.55 | 100 | 951.56 | 183 |
| 1000 | aspS | 1,359.75 | 100 | 1,116.75 | 213 |
| 1001 | rpsO | 1,364.28 | 15 | 1,052.09 | 30 |
| 1002 | CD16_RS03655 | 1,391.90 | 42 | 1,060.61 | 83 |
| 1003 | mdh | 1,402.53 | 55 | 1,474.89 | 150 |
| 1004 | CD16_RS05520 | 1,406.13 | 28 | 2,769.00 | 143 |
| 1005 | rplR | 1,420.66 | 21 | 1,069.48 | 41 |
| 1006 | CD16_RS04090 | 1,446.09 | 109 | 905.45 | 177 |
| 1007 | fabA | 1,466.80 | 31 | 1,277.10 | 70 |
| 1008 | CD16_RS05310 | 1,474.63 | 49 | 1,241.62 | 107 |
| 1009 | CD16_RS05420 | 1,482.91 | 25 | 1,326.55 | 58 |
| 1010 | rplV | 1,488.31 | 24 | 1,339.02 | 56 |
| 1011 | CD16_RS01115 | 1,490.02 | 79 | 1,047.24 | 144 |
| 1012 | tolB | 1,498.27 | 82 | 1,507.68 | 214 |
| 1013 | fabG | 1,518.31 | 46 | 1,259.96 | 99 |
| 1014 | CD16_RS04135 | 1,519.70 | 44 | 2,077.55 | 156 |
| 1015 | fabB | 1,528.53 | 76 | 1,783.64 | 230 |
| 1016 | dut | 1,531.51 | 29 | 1,262.51 | 62 |
| 1017 | cyoB | 1,534.82 | 126 | 1,084.97 | 231 |
| 1018 | CD16_RS04165 | 1,538.66 | 25 | 1,044.18 | 44 |
| 1019 | CD16_RS04555 | 1,539.19 | 22 | 1,133.02 | 42 |
| 1020 | CD16_RS04550 | 1,539.88 | 57 | 729.17 | 70 |
| 1021 | CD16_RS02315 | 1,539.88 | 19 | 812.51 | 26 |
| 1022 | rpoB | 1,540.35 | 261 | 1,834.14 | 806 |
| 1023 | CD16_RS04645 | 1,552.46 | 66 | 1,487.44 | 164 |
| 1024 | rplQ | 1,553.49 | 26 | 1,336.23 | 58 |
| 1025 | rplJ | 1,561.43 | 33 | 1,477.79 | 81 |
| 1026 | CD16_RS01765 | 1,584.33 | 24 | 1,323.60 | 52 |
| 1027 | CD16_RS03105 | 1,595.95 | 62 | 1,052.09 | 106 |
| 1028 | CD16_RS00445 | 1,599.50 | 102 | 1,233.49 | 204 |
| 1029 | clpX | 1,617.88 | 84 | 1,945.75 | 262 |
| 1030 | CD16_RS05485 | 1,620.52 | 117 | 1,548.76 | 290 |
| 1031 | ndk | 1,625.53 | 28 | 1,365.48 | 61 |
| 1032 | rpmE | 1,637.14 | 15 | 1,094.17 | 26 |
| 1033 | fabD | 1,637.14 | 63 | 1,773.52 | 177 |
| 1034 | yacG | 1,662.72 | 13 | 2,317.89 | 47 |
| 1035 | CD16_RS04110 | 1,663.49 | 101 | 1,111.36 | 175 |
| 1036 | grpE | 1,674.34 | 45 | 3,314.09 | 231 |
| 1037 | fusA | 1,679.11 | 144 | 1,686.04 | 375 |
| 1038 | rpsH | 1,700.10 | 27 | 2,233.67 | 92 |
| 1039 | rpsU | 1,705.35 | 20 | 2,432.96 | 74 |
| 1040 | CD16_RS03360 | 1,713.28 | 18 | 2,055.25 | 56 |
| 1041 | CD16_RS03940 | 1,731.59 | 22 | 789.07 | 26 |
| 1042 | rpsR | 1,754.07 | 18 | 2,667.80 | 71 |
| 1043 | CD16_RS02650 | 1,802.35 | 24 | 2,750.88 | 95 |
| 1044 | dnaJ | 1,807.23 | 85 | 1,754.40 | 214 |
| 1045 | rplU | 1,810.29 | 23 | 2,579.65 | 85 |
| 1046 | CD16_RS00720 | 1,810.66 | 48 | 1,861.76 | 128 |
| 1047 | rpsC | 1,831.01 | 51 | 1,688.88 | 122 |
| 1048 | CD16_RS02710 | 1,849.65 | 47 | 849.77 | 56 |
| 1049 | map | 1,855.03 | 63 | 1,487.31 | 131 |
| 1050 | CD16_RS04870 | 1,903.65 | 20 | 1,688.24 | 46 |
| 1051 | CD16_RS04290 | 1,932.73 | 51 | 2,104.18 | 144 |
| 1052 | rpsD | 1,937.67 | 49 | 1,540.02 | 101 |
| 1053 | CD16_RS03835 | 1,939.73 | 50 | 1,735.20 | 116 |
| 1054 | rpsK | 1,951.97 | 31 | 825.49 | 34 |
| 1055 | CD16_RS00210 | 1,956.93 | 82 | 2,236.08 | 243 |
| 1056 | rplS | 1,959.95 | 34 | 1,800.41 | 81 |
| 1057 | dnaA | 1,985.39 | 122 | 1,644.02 | 262 |
| 1058 | rplW | 1,991.11 | 27 | 1,905.14 | 67 |
| 1059 | pnp | 2,023.03 | 173 | 1,772.02 | 393 |
| 1060 | rplT | 2,046.42 | 31 | 1,374.51 | 54 |
| 1061 | CD16_RS01475 | 2,055.76 | 55 | 2,060.95 | 143 |
| 1062 | CD16_RS04155 | 2,057.60 | 46 | 1,914.46 | 111 |
| 1063 | fabF | 2,065.73 | 107 | 1,377.15 | 185 |
| 1064 | CD16_RS05275 | 2,067.96 | 72 | 2,680.06 | 242 |
| 1065 | trpS | 2,115.40 | 92 | 1,471.75 | 166 |
| 1066 | CD16_RS01460 | 2,174.32 | 51 | 3,304.22 | 201 |
| 1067 | CD16_RS04160 | 2,196.16 | 33 | 1,590.97 | 62 |
| 1068 | greA | 2,265.22 | 44 | 2,124.03 | 107 |
| 1069 | CD16_RS05230 | 2,284.38 | 24 | 1,541.44 | 42 |
| 1070 | CD16_RS02030 | 2,290.29 | 54 | 3,826.78 | 234 |
| 1071 | CD16_RS03210 | 2,338.77 | 20 | 1,172.33 | 26 |
| 1072 | CD16_RS04395 | 2,338.77 | 36 | 2,029.03 | 81 |
| 1073 | gyrA | 2,417.07 | 269 | 2,016.41 | 582 |
| 1074 | CD16_RS00965 | 2,425.39 | 56 | 2,087.48 | 125 |
| 1075 | CD16_RS05235 | 2,447.93 | 189 | 1,992.65 | 399 |
| 1076 | rpsF | 2,588.12 | 43 | 2,970.61 | 128 |
| 1077 | bamE | 2,762.67 | 54 | 3,669.17 | 186 |
| 1078 | CD16_RS05225 | 2,798.97 | 53 | 2,606.47 | 128 |
| 1079 | CD16_RS03675 | 2,800.36 | 39 | 1,605.82 | 58 |
| 1080 | CD16_RS05555 | 2,847.19 | 40 | 1,811.43 | 66 |
| 1081 | CD16_RS03095 | 3,114.12 | 35 | 2,538.74 | 74 |
| 1082 | infA | 3,244.77 | 44 | 2,985.66 | 105 |
| 1083 | rpsI | 3,255.12 | 68 | 3,783.84 | 205 |
| 1084 | CD16_RS05535 | 3,374.25 | 54 | 4,120.02 | 171 |
| 1085 | CD16_RS00980 | 3,377.43 | 203 | 2,886.83 | 450 |
| 1086 | CD16_RS05205 | 3,410.70 | 35 | 2,517.50 | 67 |
| 1087 | rpsP | 3,428.19 | 49 | 4,693.94 | 174 |
| 1088 | ssrA | 3,471.41 | 54 | 2,280.45 | 92 |
| 1089 | CD16_RS04810 | 3,500.79 | 68 | 2,282.84 | 115 |
| 1090 | trxA | 3,562.29 | 47 | 2,513.33 | 86 |
| 1091 | CD16_RS01455 | 3,594.77 | 83 | 2,905.77 | 174 |
| 1092 | CD16_RS04755 | 3,672.59 | 67 | 2,346.07 | 111 |
| 1093 | sppA | 3,703.05 | 133 | 3,542.75 | 330 |
| 1094 | CD16_RS03935 | 3,730.18 | 108 | 2,876.60 | 216 |
| 1095 | rplM | 3,778.01 | 72 | 4,167.90 | 206 |
| 1096 | rpsA | 3,802.01 | 268 | 2,696.78 | 493 |
| 1097 | CD16_RS02255 | 3,866.86 | 77 | 4,008.27 | 207 |
| 1098 | CD16_RS03945 | 3,914.89 | 44 | 1,612.44 | 47 |
| 1099 | CD16_RS03900 | 4,171.55 | 106 | 3,490.11 | 230 |
| 1100 | rplL | 4,253.98 | 66 | 3,454.50 | 139 |
| 1101 | CD16_RS04750 | 4,425.98 | 93 | 5,358.32 | 292 |
| 1102 | CD16_RS03555 | 4,490.20 | 113 | 4,259.44 | 278 |
| 1103 | CD16_RS04385 | 4,858.17 | 73 | 3,284.58 | 128 |
| 1104 | CD16_RS04035 | 4,973.58 | 48 | 5,513.49 | 138 |
| 1105 | CD16_RS00940 | 5,371.85 | 231 | 6,348.41 | 708 |
| 1106 | CD16_RS00915 | 5,564.42 | 121 | 3,475.45 | 196 |
| 1107 | dnaK | 5,588.06 | 441 | 7,221.32 | 1478 |
| 1108 | rpsT | 6,296.68 | 70 | 4,855.80 | 140 |
| 1109 | CD16_RS02375 | 6,462.38 | 60 | 2,948.62 | 71 |
| 1110 | CD16_RS03695 | 7,381.73 | 101 | 8,736.11 | 310 |
| 1111 | CD16_RS01625 | 8,437.55 | 268 | 8,643.33 | 712 |
| 1112 | CD16_RS04895 | 8,731.39 | 64 | 5,681.29 | 108 |
| 1113 | CD16_RS00895 | 8,865.46 | 313 | 8,474.97 | 776 |
| 1114 | groL | 9,327.52 | 629 | 13,517.08 | 2364 |
| 1115 | CD16_RS05110 | 9,562.03 | 264 | 8,449.31 | 605 |
| 1116 | CD16_RS02445 | 9,922.04 | 200 | 7,039.44 | 368 |
| 1117 | CD16_RS04885 | 13,067.05 | 174 | 9,642.56 | 333 |
| 1118 | CD16_RS04875 | 18,626.60 | 223 | 18,969.84 | 589 |
| 1119 | CD16_RS04880 | 19,157.98 | 220 | 20,012.11 | 596 |
| 1120 | CD16_RS02370 | 19,189.38 | 143 | 14,125.61 | 273 |
| 1121 | CD16_RS02920 | 21,552.17 | 416 | 16,720.25 | 837 |
| 1122 | CD16_RS03055 | 23,802.05 | 599 | 22,706.78 | 1482 |
| 1123 | CD16_RS04890 | 25,116.31 | 494 | 13,977.78 | 713 |
| 1124 | CD16_RS05425 | 34,549.22 | 816 | 39,948.61 | 2447 |
| 1125 | rnpB | 50,262.21 | 788 | 26,242.15 | 1067 |

^a^ The order of genes was sorted from smallest to biggest based on CLas genes TPM of citrus HiSeq data.
